# Three-Dimensionally Printed Self-Lock Origami: Design, Fabrication, and Simulation to Improve Performance of Rotational Joint

**DOI:** 10.3390/mi14081649

**Published:** 2023-08-21

**Authors:** Samira Zare, Alex Spaeth, Sandya Suresh, Mircea Teodorescu

**Affiliations:** 1 Department of Electrical and Computer Engineering, University of California Santa Cruz, Santa Cruz, CA 95064, USA; atspaeth@ucsc.edu; 2 SIP Program, University of California Santa Cruz, Santa Cruz, CA 95064, USA; sandya.suresh@gmail.com

**Keywords:** origami, rotational joint, deployable structure, modular, flat-foldable, bidirectional joint, self-lock, 3D printing, pouch motor, manipulator

## Abstract

Origami structures have made significant contributions to the field of robotics, offering various advantages. One such advantage is their ability to conserve space by transforming the structure into a compact form. Additionally, many origami structures can be fabricated in a flat state to simplify manufacturing, giving them the potential for large-scale and cost-effective production. Rotational joints play a crucial role in the construction of robotic systems, yet origami rotational joints can suffer from a limited range of motion. We previously theoretically proposed the Self-Lock Joint to address this issue, but it is only partially flat-foldable. This paper presents a novel approach to the 3D printing of modular origami joints, such as the Self-Lock Joint, using 3D-printed plates joined with a fabric layer. The compliance of the fabric can improve the joint’s semi flat-foldability or even enable it to achieve complete flat-foldability. Furthermore, the rotational motion of the joint is enhanced, allowing for close to 360 degrees of rotational movement. We assess the physical properties of the joint under both loaded and unloaded conditions in order to identify design trade-offs in the physical properties of the joints. Moreover, as a proof of concept, we construct and demonstrate manipulators utilizing these joints. The increase in rotational movement enabled by this fabrication method, coupled with the compliant joint’s flat-foldability and modular nature, make it a promising candidate for use in a wide range of applications.

## 1. Introduction

Origami is an ancient art of paper folding that has seen significant use in modern robotics [1]. The numerous advantages of origami structures include adaptability to their surroundings [2,3] and the potential for fast and cost-effective production [4,5]. Furthermore, many origami structures can either transform into flat sheets or fold into compact forms, simplifying manufacturing and deployment [6].

Three-dimensional printing, also known as additive manufacturing, offers numerous advantages that have revolutionized various industries. One key advantage is its ability to create complex geometries and intricate designs with high precision, enabling the production of customized and personalized objects [7]. Additionally, 3D printing allows for rapid prototyping, reducing the time and cost involved in product development. It also enables on-demand manufacturing, eliminating the need for extensive storage and inventory. Furthermore, 3D printing promotes sustainability by minimizing material waste through precise layer-by-layer construction. With its versatility, speed, cost-efficiency, and design flexibility, 3D printing has emerged as a promising method for creating origami structures.

The integration of 3D printing technology with the art of origami combines their advantages, offering an effective structure for different applications. Researchers have used various methods to create 3D-printed origami. Abstractly, origami consists of solid parts (origami plates) and soft parts (foldlines). These soft and solid parts can be fabricated separately and manually integrated to create the structure [8]. Such structures can carry heavy loads, but this fabrication method requires manual labor, which increases production time and cost, as well as presents challenges for fabricating small or complex structures.

Another method involves embedding rigid origami structures in a fabric skin (soft material) using lamination [9,10,11] or by employing an adhesive layer [12]. The use of fabric adds high flexibility and low weight, and enables foldable motion in the structure. However, this method has difficulty fabricating very small, delicate, or thin origami structures. Additionally, multimaterial 3D printing is widely employed for 3D-printed origami by fabricating a rigid core in a rubber-like frame made of viscoelastic material [13,14,15,16,17,18]. Finally, it is possible to create structures from a single compliant material, using mechanical furrows to ensure that creases form at desired locations [19,20]. This is more similar to traditional single-material (paper) origami and can approximate the benefits of multimaterial printing on much cheaper hardware.

Four-dimensional printing techniques [20,21,22,23,24] are one of the branches of multimaterial printing. Using 4D printing techniques makes it possible to create 3D objects that exhibit morphing and mechanical behaviors, all meticulously programmed within the material architecture. This advancement brings us closer to uncovering the design principles underlying the mesmerizing dynamics of origami’s self-shaping structures. A remarkable example of this is the earwig-inspired spring origami joint, designed by Faber et al. [25]. This joint’s flexibility and extensibility enable it to achieve self-folding and self-locking mechanisms. However, non-rigid origami comes with its inherent trade-offs. The joint spring must be precisely tailored to the specific task at hand, and over time, the stretchability of the fold lines naturally diminishes with repeated use. Moreover, depending on the number of involved folds, the width of the flexible fold lines might increase, leading to a potential compromise in structural strength.

Three-dimensionally printing both soft and solid materials simultaneously offers fast and cost-effective fabrication. This advantage can even be extended to new combinations of materials by combining multiple additive manufacturing technologies together [26]. In particular, using fabric as the soft material offers even higher flexibility, reduced thickness, and lighter weight for the soft joint. Therefore, the integration of 3D printing on fabric is a significant area of investigation in various fields, including fashion [27,28,29], medical textiles [30,31], and wearable technology [32,33,34]. Three-dimensional printing has been used to create smart wearables by integrating push buttons directly onto fabrics [35,36]. Furthermore, researchers have leveraged the capabilities of 3D printing, combined with Shape-Memory Alloys, to develop customizable actuators [37]. However, 3D printing on fabric has not been extensively utilized in the fabrication of origami joints.

Rotational motion serves as a fundamental joint structure in many robotic systems. Therefore, developing a flat-foldable origami joint with rotational capabilities would offer significant benefits to robotics. Previous research has proposed the Four-Vertex Joint [38], an origami joint based on the Miura-ori unit cell that greatly enhances the range of rotational motion available when using a pneumatic actuator called pouch motors [39,40]. However, this joint had a degenerate configuration, which could lead to actuation failure, so recently, we proposed the Self-Lock Joint [41], a modification of the Four-Vertex Joint that always has one degree of freedom thanks to the self-locking property. Self-locking is a special type of structural rigidity specific to origami structures [42,43]. Origami with self-locking behavior can exhibit complex motions particularly suited to modular robotic applications [19,20]; indeed, the Self-Lock Joint was demonstrated in the context of modular manipulators. However, the Self-Lock Joint was designed as a theoretical rigid origami model, whose self-locking property prevented the joint from reaching its flat-folded state [41]. The focus of this paper is to utilize 3D printing on fabric to address this limitation and to design a fabrication method for achieving complete flat-foldability in origami joints.

The contributions of this paper include the following:

- Proposing two 3D printing methods for creating the Self-Lock Joint;
- Designing and developing an activation system for the joints;
- Analyzing the performance of the origami joints with various physical properties;
- Developing an origami joint mechanism for bi-directional movement;
- Developing both bi-directional and one-directional manipulators utilizing the designed joints.

## 2. Materials and Methods

**Figure 1:**
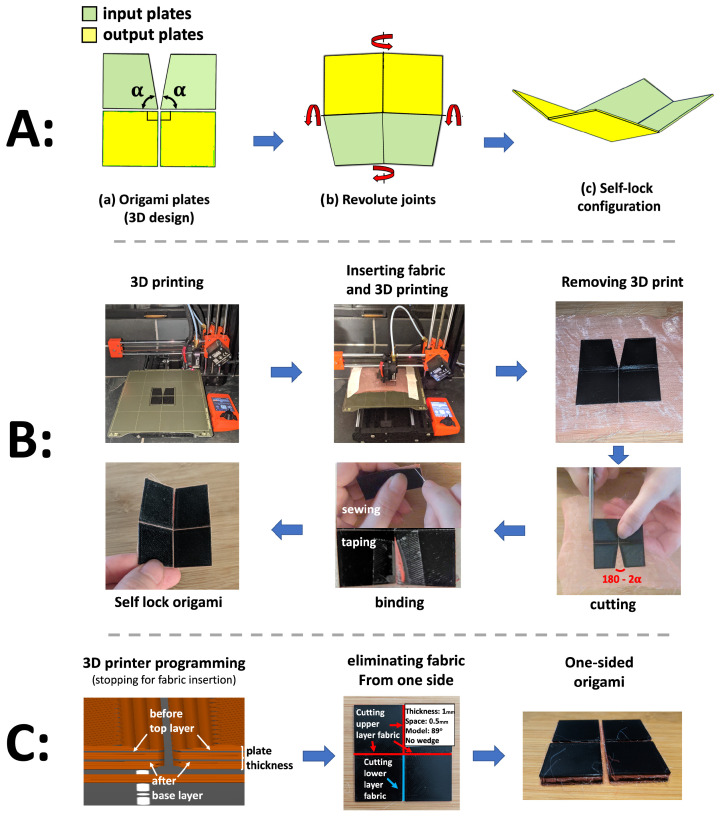
(**A**) CAD model of the Self-Lock Joint. (**a**) The origami plates are designed in a single plane so that fabric can easily be inserted during printing. The reduced central angles are denoted by α. The joint consists of two plates actuated using a pouch motor, referred to as input plates (green), and two “output” plates that move in response (yellow). (**b**) In simulation, the plates are brought together and joined via revolute joints. (**c**) Three-dimensional view of the joint. Due to its reduced central angles, the rigid model cannot achieve a flat state. (**B**) For the the bidirectional joint, printing paused halfway through to allow for the insertion of fabric, which is secured with tape. After removal from the printer, the external fabric surrounding the origami is cut. Then, in the binding phase, the internal fabric between the reduced angles of the origami plates (180−2α) is taped or sewn. (**C**) The 3D printing method with dual fabric layers is similar, but printing is paused twice so that two layers of fabric can be inserted. In the “eliminating fabric” phase, the upper layer of fabric is cut along the red fold lines, while the bottom layer of fabric is cut along the blue fold lines.

We developed two methods to fabricate the Self-Lock Joint using 3D printers. Both begin with designing a CAD model of the origami plates (Figure 1A(a)). The origami consists of two “input” plates with central angle α, which are actuated, as well as two “output” plates with central angle $90^{\circ }$. In the CAD, mate constraint relationships are defined between the plates to ensure that they all lie in the same plane (flat state) for 3D printing. Furthermore, a variable gap is defined between the origami plates using the constraint relationship, except for the location of the cutting angles for the Self-Lock Origami. Figure 1A(b,c) show the directions of movements and configuration of the Self-Lock Joint in the dynamic simulation environments.

**Figure 2:**
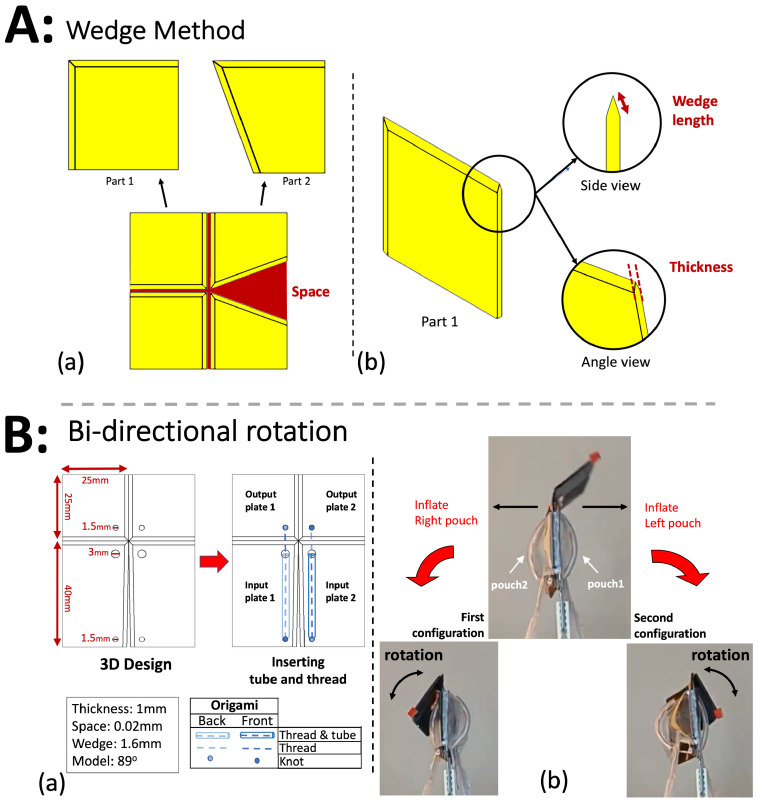
(**A**) The bidirectional origami incorporates wedges along the edges of adjacent origami plates. (**a**) The wedge design is implemented for all four origami plates. Although the origami consists of four plates, by symmetry, there are only two distinct parts. (**b**) A detailed examination of the wedge design is provided in side and angle perspectives. The size and thickness of the plates are indicated in red. (**B**) The bidirectional origami joint. (**a**) Schematic of tubes and threads on the input and output origami plates. (**b**) When the right pouch inflates, it pulls the thread and tube, causing the origami to transition to the first configuration (rotating left). The right rotation operates in a similar manner.

The first printing method we propose is shown in Figure 1B. The CAD model is exported to STL format for 3D printing with a PLA filament. The plates are printed directly on the print bed. The software PrusaSilcer 2.6.0 is used to program the Prusa 3D printer to pause printing while a layer of fabric is inserted to act as the middle layer of the origami. The gaps between the plates are filled with fabric to add flexibility and a small amount of extensibility. This allows the origami to fold beyond the limits of the rigid model, while still obtaining a strong structure similar to a traditional revolute joint. Clips and tapes are used to hold the fabric in place on the 3D printer plate while the plates are being printed. After the 3D printer job is finished, we remove the origami and its embedded fabric from the print bed and then trim the excess fabric surrounding the joint. The fabric in the triangular gap created by the reduced central angles of the Self-Lock Joint is cut and then recombined by hand with a needle and thread. For more efficient manufacturing, this could be performed with tape or by melting. Due to the high flexibility and slight extensibility of fabric, the Self-Lock Origami with α close to 90${}^{\circ }$ is capable of being flattened completely. This enables both flat-foldability (e.g., for storage) and bidirectional movement (Figure 2B(b)).

Figure 1C illustrates an alternative method for origami 3D printing using dual-layer fabric insertion. In this approach, we incorporated two pauses during the 3D printing process using the PrusaSlicer software. The first pause occurs after printing the base layer of the origami plates, while the second pause takes place prior to printing the final layer of the plates. During each pause, a sheet of fabric is inserted and secured with tape.

The subsequent steps involving 3D printing removal, cutting, and binding are similar to the previous method described in Figure 1B. However, the top and bottom fabrics between the plates are cut based on the desired configuration and movement of the origami. For the Self-Lock Joint to exhibit a downward rotation, three of the fold lines should rotate in one direction, while the remaining fold line should rotate in the opposite direction [38]. Consequently, the top layer fabric of three foldlines between the origami plates (highlighted in red in Figure 1C, eliminating fabric), and the bottom layer of fabric between the remaining origami plates (shown in blue) is removed. As a result, a 3D-printed origami structure with fabric fold lines is achieved, capable of rotating in a single direction.

### 2.1. Pouch Actuators

The activation of the joint is achieved by installing pneumatic pouches [39] made of vinyl sheet around the input plates. The pouches are 25 mm × 4.9 mm square, which are attached to the input plates of the origami on either end, such that when they are inflated, they contract and pull the input plates together. Two types of pouches have been developed for different applications. Type A pouches feature connectors at the bottom, while Type B pouches have connectors on the side. The pouches are otherwise identical (see Figure S1B(e)). This differentiation is particularly advantageous in bidirectional origamis used in manipulators. When the Type A pouch’s connector and tube would obstruct the rotation of another origami, the Type B pouch can be employed to overcome this limitation.

Each origami joint is actuated via two antagonistic pouch motors. We control the pouch pressure using a single pump, with the airflow to a particular pouch determined by solenoid-driven air valves controlled using a microcontroller. The control strategy is bang-bang, with pressure sensors used for safety. This system is described in more detail in Section S1.

### 2.2. Wedge Method

As the thickness of the origami plates increases or the length of fabric between the plates decreases, the joint becomes stronger. This is discussed in more detail in Section 3. However, these changes can also impose constraints on the movement of the origami joint. This does not affect the movement of the origami in the second 3D printing method using dual layer fabric because the fabric is positioned very close to the surface of the plate, but it can limit the range of motion of origami constructed according to the first method. To address this issue, we create a wedge-shaped profile along the edges of adjacent origami plates. Figure 2A illustrates these wedges on the origami plates. This wedge has a single parameter, labeled “wedge length” in Figure 2A(b), whose influence on the design is studied in Section 3.2 and Section 3.3.

### 2.3. Bidirectional Origami

The origami model has a plate thickness of 1 mm and a wedge length of 1.6 mm. The space between the plates is 0.02 mm, although this may vary due to 3D printer precision and the compliance of the fabric. It is vertically symmetrical, featuring 25 mm × 25 mm output plates and 25 mm × 40 mm input plates. The central angle of the input plates is then reduced to an angle $\alpha <90^{\circ }$ by cutting a triangular section from the inside edge of each input plate [41]. This is the physical Self-Lock Joint, which we use for most of our experiments.

The theoretical rigid Self-Lock Joint has two configurations, which rotate in opposite directions, but it cannot transition between them without being reassembled [41]. To achieve both configurations using only two pouch actuators on both sides of the origami [38], we include threads on top of each pouch, which pull the output plates towards the pouch, which inflates first. The result is an origami joint that can rotate in either direction, which we call the bidirectional joint.

Figure 2B(a) showcases the 3D design of the origami, as well as the placement of the threads and tubes. In the CAD model, 1.5 mm and 3 mm holes are incorporated for the insertion of threads, tubes, and knots. Knots at the bottom and top holes of the origami pull the threads when the pouches inflate. Tubes are necessary to spread the load of the thread across more of the pouch so that they interfere minimally with inflation. It is also necessary to include some slack in these tubes to avoid interfering with the compliance of the structure.

Figure 2B(b) illustrates the functionality of the bidirectional origami. When the right pouch inflates, it pulls the tube and the thread, causing the origami to rotate left and transition to the first configuration. Additionally, the inflation of the pouch decreases its length [39], which leads to the input plates around it rotating toward each other, while the output plates rotate to the left. Similarly, when the left pouch inflates, the output plates rotate to the right.

One limitation of this approach is that although the origami is kinematically capable of almost 360${}^{\circ }$ of rotation between the two directional configurations [41], in practice, it runs into the pouches and tubes. We investigate this limitation in Section 3. Furthermore, the force generated by the threads is not sufficient to support heavy loads. Therefore, in applications where a strong structure is required, it is advisable to employ a second actuator instead of threads and tubes.

### 2.4. Experiment Setup

In all experiments, one of the input plates is grounded, and a marker is placed at the outer corner of the output plate, which is connected to the grounded input plate. The coordinate reference frame is defined with the *x* axis along the horizontal edge of the grounded plate and the *y* axis along the vertical edge. In some experiments, a weight of 49 g is applied to the marker’s plate to evaluate the structural integrity and capabilities of the joint. The physical setups are shown in Section 3.

The software Kinovea-0.9.5 was used to perform motion analysis in the *y*–*z* plane. The variable φ represents the angle between the grounded plate and the marker’s plate. As the joint folds to the right, φ moves down from $180^{\circ }$ towards zero, whereas a fold to the left takes φ from $-180^{\circ }$ up towards zero. A video capturing the motion of the joint is recorded, with the camera’s optical axis positioned orthogonal to the *y*–*z* plane of the joint. After calibrating the system using the grounded plate’s edge length, the marker of interest, a small red square, can be tracked using Kinovea software. Data are collected for 20 cycles of the joint’s rotational movement, including the φ values for both the folded and unfolded states of the joint, as well as the *y* and *z* position data.

Many of our experiments are compared with kinematic simulations carried out in Autodesk Inventor. The origami plates are assumed to be solid, and instead of foldlines, revolute joints are defined. Furthermore, the plates are assumed to have zero thickness. This assumption is necessary because the simulation does not consider the presence of the soft fabric foldline, which provides the necessary flexibility for the origami structure. To simulate the behavior of the origami joint, one of the joint’s input plates is grounded. A trace, representing the joint’s marker, is positioned at the outer corner of the output plate of the origami. An imposed rotational angle is applied to the rotational joint, which represents the fold line defining the angle φ. For a more detailed explanation of the motion simulation, please refer to [41].

## 3. Results

This section presents the results of experiments conducted to explore the effect of the various parameters of an origami joint design. In each case, the trajectories of a motion capture marker attached according to Section 2.4 are depicted in the *y*–*z* plane. Trajectories from folding and unfolding motions are combined into a single plot and annotated with extremal values of φ. Each origami model is constructed using the base parameters described in Section 2.3, except for the parameters that are varied in a particular experiment. All results in this section except Section 3.5 use the Self-Lock Joint (single fabric layer, no threads).

### 3.1. Model Analysis

**Figure 3:**
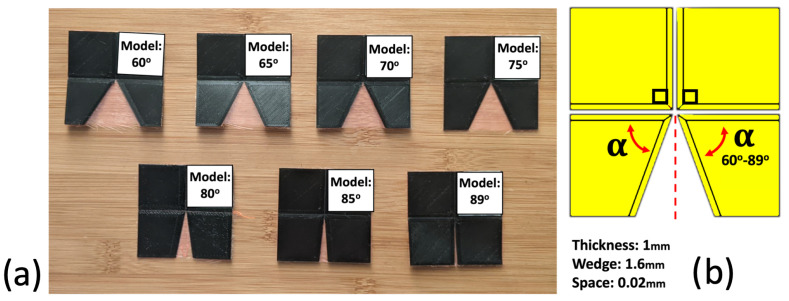
(**a**) Three-dimensionally printed Self-Lock Joints with α ranging from 60${}^{\circ }$ to 89${}^{\circ }$. (**b**) Diagram of the origami joint, which comprises two input plates with a fixed central angle 90${}^{\circ }$ and two output plates with reduced central angle α.

**Figure 4:**
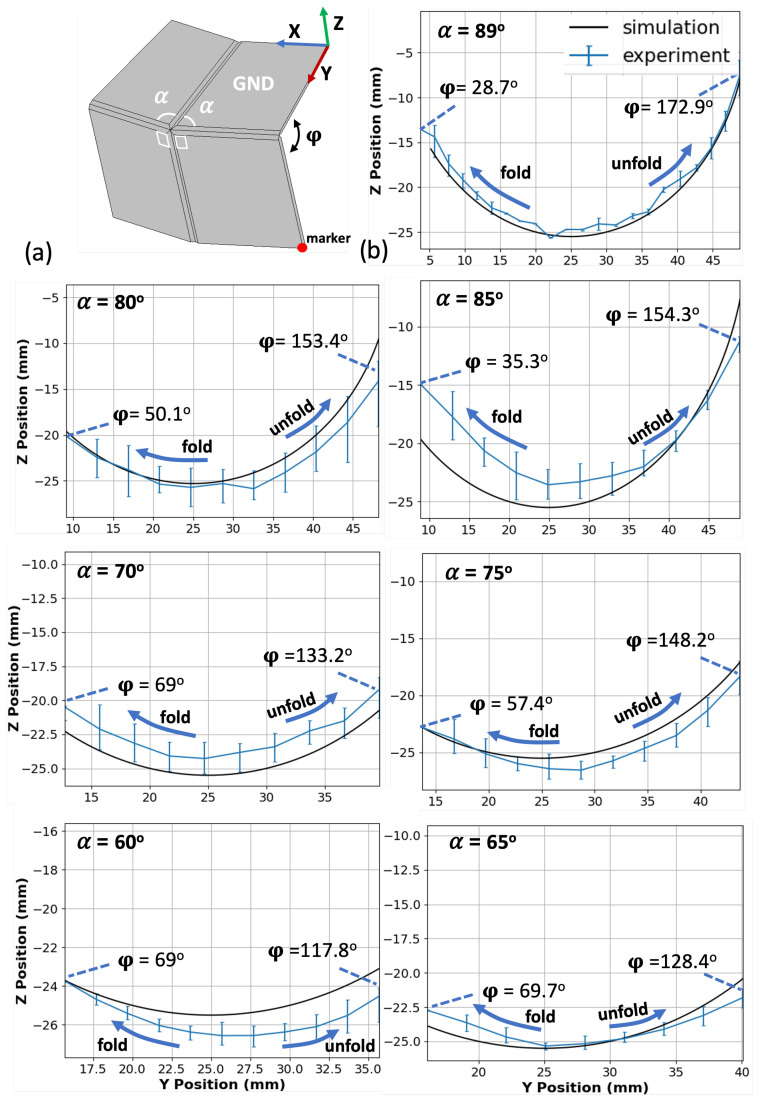
(**a**) CAD model of the experimental setup. (**b**) Trajectories of both simulated and 3D-printed origami joints with α ranging from 89${}^{\circ }$ to 60${}^{\circ }$. The folding motion proceeds from center to left, while the unfolding motion proceeds from center to right. As α decreases, the range of motion decreases as well, with the maximum value of φ decreasing and its minimum value increasing.

To study the effect of varying central angles on the motion of the Self-Lock Joint, we printed joints with different values of the reduced central angle α (Figure 3). Figure 4 illustrates the rotational motion of the markers. Both simulation and experimental data show a drastic decrease in the range of motion as the central angle α decreases from 89${}^{\circ }$ to 60${}^{\circ }$. The maximum of the output angle φ increases from 28.7${}^{\circ }$ to 69${}^{\circ }$, while its maximum decreases from 172.9${}^{\circ }$ to 117.8${}^{\circ }$. Therefore, selecting the origami joint with a higher central angle α enhances the motion performance of the joint. However, it is important to consider a trade-off when designing the joint. Values of α close to 90${}^{\circ }$, coupled with the extensibility of the fabric at the fold line, can cause the joint to change its configuration and direction of movement when subjected to large forces or when required to carry a heavy load. This trade-off must be taken into account during the design process to ensure stability of the joint for reliable performance under various operating conditions. In the following sections, we take advantage of the superior kinematics offered by $\alpha =89^{\circ }$ for unloaded experiments and set $\alpha =80^{\circ }$ in experiments involving a load. The study of one-directional Self-Lock Origami with $89^{\circ }$ degrees central angles can be found in the Supplementary Materials. The details of the mathematical model are explained in detail in [41].

### 3.2. Thickness and Wedge Analysis

**Figure 5:**
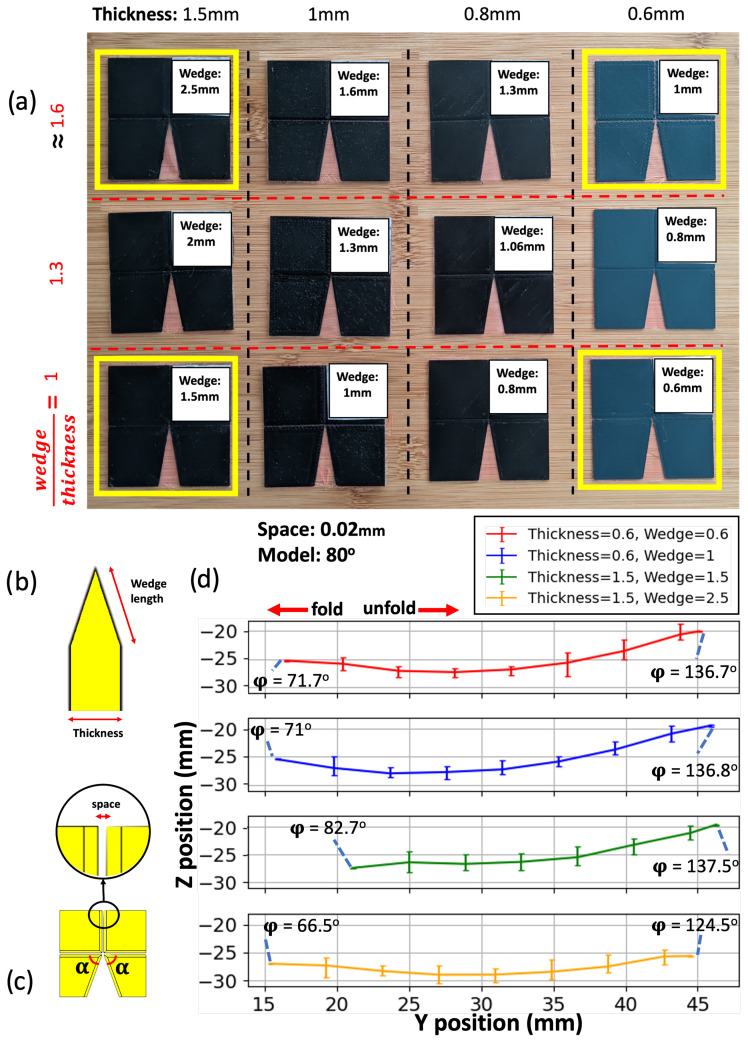
(**a**) Three-dimensionally printed Self-Lock Joint with $\alpha =80^{\circ }$, shown before the binding step, with varying plate thickness and wedge length. Rows are organized by approximate ratio of wedge length to thickness. (**b**) Diagram of the wedge length and thickness in a side view of the origami plate. (**c**) Top view diagram of the origami, highlighting the space between the plates as well as the reduced central angle α. (**d**) Trajectory of the corner of the output plate (see text), also indicating the output angle φ at the extrema of the motion.

We next studied the motion of 3D-printed Self-Lock Joint with $\alpha =80^{\circ }$, focusing on the effect of different origami plate thicknesses (Figure 5b) while maintaining a constant fabric width (Figure 5c, space) between the plates. Our objective was to create a stronger joint capable of carrying loads. However, we observed that increasing the plate thickness could potentially limit the range of movement. To address this limitation, we also explored variations in the wedge length (Figure 5b) of the origami plates while keeping the thickness constant. The aim was to determine if wedge length and plate thickness parameters would help maximize the joint motion range while still accommodating load-bearing capabilities.

**Figure 6:**
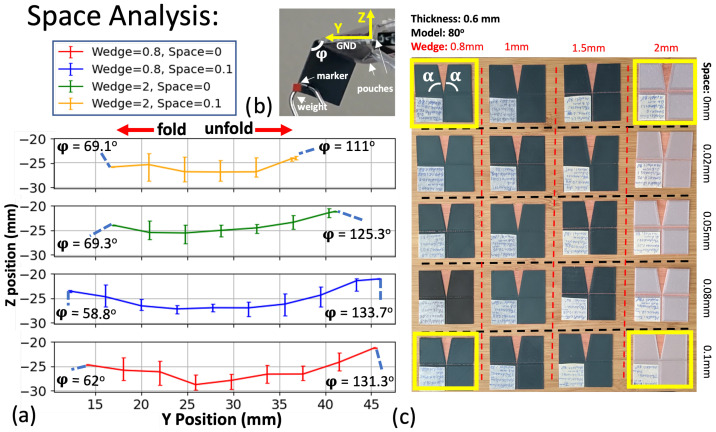
(**a**) Marker trajectories for the joints indicated in yellow in part (**c**). The folding motion occurs from center to left, while the unfolding motion occurs from center to right. (**b**) Experimental setup, highlighting the coordinate system, pouch actuators, 49 g weight, and motion capture marker. (**c**) Three-dimensionally printed Self-Lock Joints with varying wedge and space lengths. The joints with extreme parameters, which are highlighted in yellow, are selected for further analysis and study.

Figure 5a showcases the joints with different thicknesses and wedge length to thickness ratios. Wedge length and thickness were set to round-number values in CAD, resulting in the approximate wedge-length-to-thickness ratios depicted in the figure. The origami joints highlighted in yellow, with thicknesses of 0.6 mm and 1.5 mm, and wedge lengths of 0.6 mm, 1 mm, 1.5 mm, and 2.5 mm, were subjected to an experiment with a 49 g load suspended from their marker’s plates (Figure 6b).

Figure 5d illustrates the rotational motion of the joints in a *y*–*z* plot. From right to left, the concave lines indicate the folding motion of the origami, while from left to right, they represent the unfolding motion. It is observed that the range of motion is reduced compared with the $\alpha =80^{\circ }$ Self-Lock Origami without any loads (Figure 4b). As the thickness of the origami plates increases, their folding range of motion also increases. However, if the wedge length does not increase proportionally with the thickness, it can interfere with the movement and limit the folding range compared with joints with smaller thicknesses.

For unfolding, a slight increase in the range of motion is observed as the plate thickness increases with the wedge-length-to-thickness ratio held fixed. However, when the ratio of wedge length to thickness increases, the unfolding range of motion decreases. This is because the plates become too thin at the fold lines, creating a larger soft area between the solid plates, which hinders rotation when carrying a heavy load. In folding motions, the soft area between the joint’s plates decreases as the movement progresses due to the structure’s configuration. Therefore, there is a trade-off between selecting a joint with a larger thickness and wedge length. For joints that have initially small plate thicknesses (e.g., 0.6 mm), they already possess a substantial range of motion to effectively carry a load of 49 g. In such cases, the impact of varying the wedge length on their motion range is minimal. This is attributed to their lightweight nature, which allows them to maintain their mobility and flexibility to carry small loads.

### 3.3. Space and Wedge Analysis

To investigate the effect of different space lengths (Figure 5c) on the joint’s motion while carrying a load, we designed 3D-printed origami joints with varying gaps between their plates (Figure 6c). However, the space length interacts with the wedge length to determine the kinematic range of motion, so we additionally varied the wedge length. For this study, we selected a thickness of 0.6 mm and a reduced central angle of $\alpha =80^{\circ }$. We specifically focused on the extreme cases (highlighted in yellow) to analyze the effects.

Figure 5b illustrates the experimental setup for the analyses. To identify the joint parameters that maximize the rotational motion along the fold line between the grounded plate and the marker’s plate, we plotted the *y*–*z* motion curves in Figure 6a for the joints with extreme parameters, including wedge lengths of 0.8 mm and 2 mm, and space lengths of 0 mm and 0.1 mm. For the 0 mm space length, the origami can still fold because a small but non-zero space between the origami plates is created by the wedges.

In Figure 6a, the smallest range of motion under load belongs to the origami with the largest wedge length and plate space. This can be attributed to the larger soft area between the plates, leading to reduced structural integrity and stiffness at the fold lines and impeding rotational movements. Even in the folded state, the geometry of the origami joints cannot fully compensate for the presence of a large soft area at the fold lines.

On the other hand, between the origami joints with a wedge length of 0.8 mm, the one with a larger space length (0.1 mm) exhibited slightly greater rotational motion. This suggests that creating a soft area that is not excessively large could be beneficial for the joints’ movements. The observed decrease in range of motion is not a kinematic change but rather the inability of the mechanical structure to support the experimental load. As such, we seek to maximize the joint’s range of motion. To do this, it is essential to carefully balance the space length and wedge length parameters. Avoiding excessively large spaces and overly long wedge lengths can help mitigate the formation of a substantial soft area, thereby improving the overall structural integrity and enabling more efficient rotational movements in the origami joints.

### 3.4. Fabric Analysis

**Figure 7:**
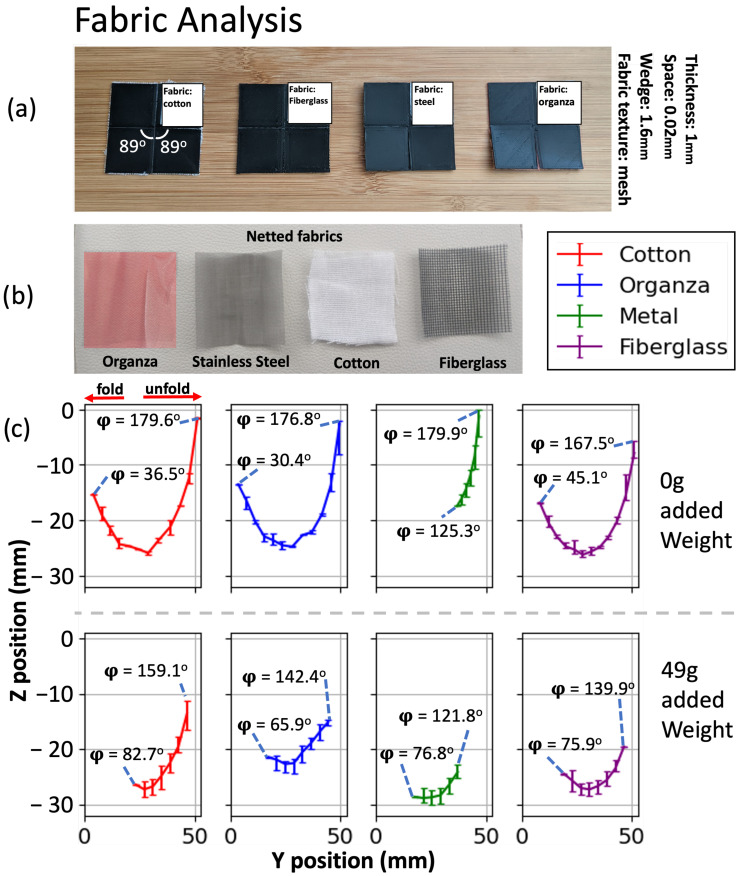
(**a**) Three-dimensionally printed Self-Lock Joints constructed using different netted fabrics as their middle layer. These joints have reduced central angle $89^{\circ }$, thickness 1 mm, wedge length 1.6 mm, and space 0.02 mm between their plates. (**b**) Samples of the fabrics used to construct the joints from part a. (**c**) *y*–*z* plots of marker trajectories as well as output angle φ for origami joints with different fabric materials as their middle layer. The top row of plots shows experiments without any load, while the bottom row shows experiments with a 49 g weight attached to the output plate.

To study the impact of different materials on the motion of origami joints, we utilized organza, stainless steel mesh, cotton, and fiberglass (Figure 7b). These four fabrics are specifically chosen as commonly available materials with a mesh texture. This texture is essential to ensure binding with the origami plates during the printing process. Figure 7a showcases 3D-printed origami joints with $\alpha =89^{\circ }$ utilizing these fabric materials.

Figure 7c shows the marker trajectory with error bars for combined folding and unfolding motions, both unloaded (top row) and with the 49 g load (bottom row). The joints without any added weight exhibit a greater range of motion. However, it is worth noting that the stainless steel material displays the smallest range of motion in both the weighted and unweighted scenarios due to its inherent stiffness. Even in the absence of any additional weight, the actuator encounters challenges in folding the netted stainless steel sheet. Despite its stiffness, the material does not enhance the movement of the marker when weight is applied.

Among the other three fabrics, organza and cotton exhibit the largest range of motion. The joint utilizing fiberglass fabric demonstrates a smaller range of motion compared with that utilizing cotton and organza when weight is added, primarily due to its lower thread count, resulting in a weaker fold line. The joint utilizing organza fabric showcases a greater folding range compared with that utilizing cotton in both the weighted and unweighted scenarios, attributed to its small stretchability properties. Conversely, cotton performs better in unfolding, although it experiences wear and tear over time. As organza fabric possesses the highest thread count, it exhibits the longest durability. Consequently, we have selected organza fabric as the optimal material for the origami joint.

### 3.5. Bidirectional Origami Joint

**Figure 8:**
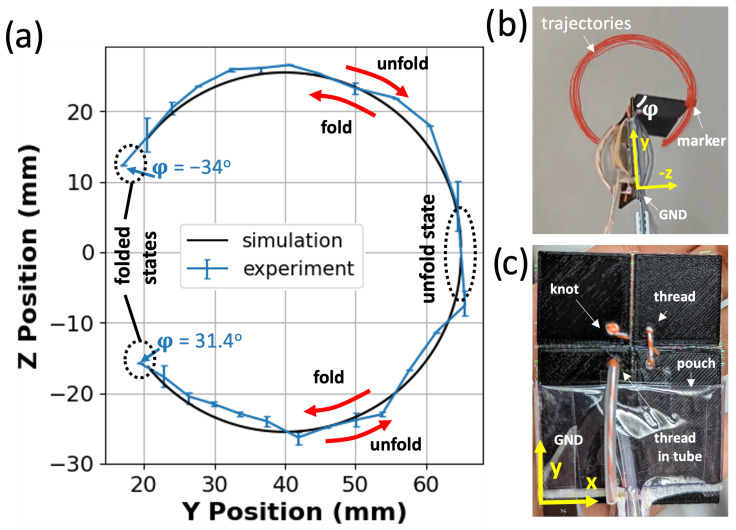
(**a**) Experimental vs. simulated motion of the marker as the bidirectional origami joint unfolds from left to right about 300${}^{\circ }$, transitioning from one folded state through the unfolded state to the other folded state. (**b**) The experiment setup for the bidirectional origami. The trajectories of the marker’s movement are represented in red. (**c**) Top view of the 3D-printed bidirectional origami showcasing the inclusion of knots, threads, threads with tubes, and pouch actuators in the experiment. The grounded plate and the reference frame are marked.

Figure 8 depicts the motion of the bidirectional joint with $\alpha =89^{\circ }$. The marker’s motion starts from one of the folded states, either on the left ($\varphi =-34^{\circ }$) or on the right ($\varphi =31.4^{\circ }$). It follows a circular path as it unfolds. This circular motion is a result of the rotation of the marker plate along the fold line of the origami joint. When the joint approaches the unfolded state ($\varphi =180^{\circ }$), it transitions from one configuration to the other using the thread and tube system: the inflation of the opposite pouch pulls the thread, which provides sufficient force to move the output plates past the unfolded state. After passing the unfolded state, the joint transitions into the folded state in the new configuration.

In the simulation, the equivalent marker follows a similar path to transition from one configuration to another. The joint experiences almost 300${}^{\circ }$ rotation between its grounded plate (input plate) and marker plate (output plate), with the two pouches serving as actuators. This motion is shown as a function of time in Figure S3 in Supplementary Materials.

## 4. Manipulators

As a proof of concept, we developed origami manipulators using the 3D-printed joints. In this section, we examine the motions of two types of 3D-printed rotational manipulators: the Bidirectional Manipulator and the Self-Lock Manipulator. Each manipulator consists of two 3D-printed origami joints with the same parameters as in Section 2.3 and that are identical to each other except that their pouch actuators are constructed with nozzles in different locations to minimize the interference of the tubing on the motion of the origami, as described in Section 2.1. In each case, Origami 1 is mounted as described in Section 2.4, but the motion capture marker is placed on the output plate of Origami 2. The output angle φ is calculated separately for the two joints and reported as $\varphi_{1}$ and $\varphi_{2}$.

**Figure 9:**
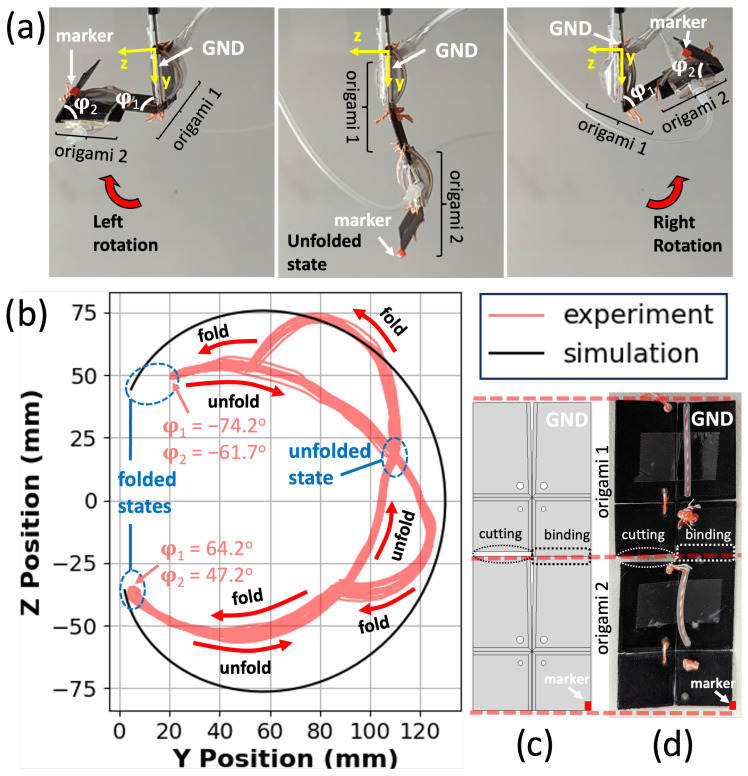
(**a**) The Bidirectional Manipulator, consisting of two bidirectional origami joints. Extrema of folding in both directions are pictured, as well as an unactuated state with both joints in their semi-flat state. The variables $\varphi_{1}$ and $\varphi_{2}$ represent the maximum folding angles of origami 1 and origami 2 respectively. (**b**) Trajectories of the manipulator marker in simulation and experiment, during folding and unfolding trajectories both left and right. The states pictured in part a are marked in blue. The printed manipulator exhibits hysteretic behavior absent from kinematic simulations; see text. (**c**,**d**) Top view of the Bidirectional Manipulator, CAD and printed versions, respectively. Red dashed lines indicate the ends of the two origami joints making up the manipulator. “Binding” indicates the connection point where the two joints meet, and “cutting” highlights the locations of cutaways in the model that prevent the two joints from interfering with each other during operation.

**Figure 10:**
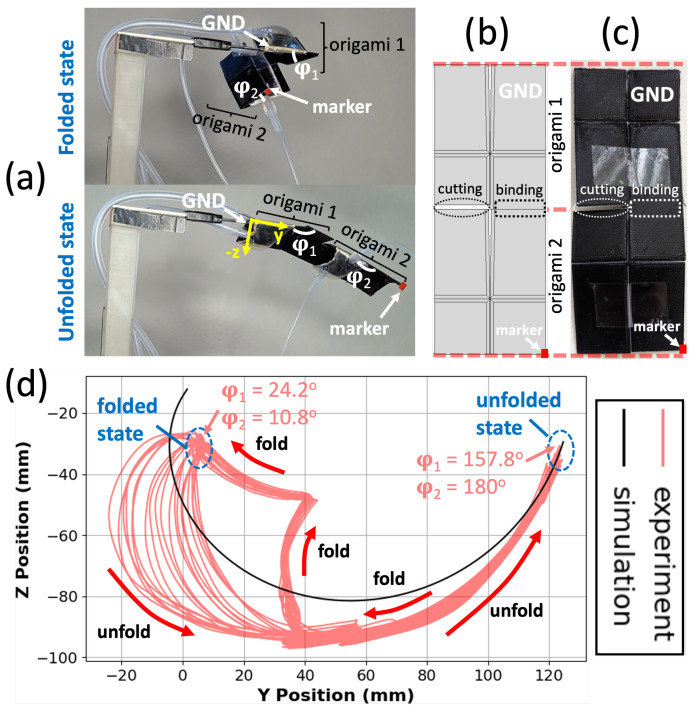
(**a**) The Self-Lock Manipulator in folded and unfolded states, consisting of two bidirectional origami joints without the threads and tubes. (**b**,**c**) Top view of the Self-Lock Manipulator, CAD and printed versions, respectively. (**d**) Trajectories of the manipulator marker in simulation and experiment. Hysteresis behavior similar to the Bidirectional Manipulator is observed; see text.

The junction between the two joints making up each manipulator is made with weld constraints in Autodesk Inventor-2023, allowing the software to treat them as one large plate. However, to prevent overconstraint of the manipulators in the CAD setting, as well as self-collisions in physical experiments, it is necessary to introduce a small cutaway to the adjacent plates of separate origami joints. These points are circled with dotted lines in Figure 9c,d and Figure 10b,c.

### 4.1. Bidirectional Manipulator

The Bidirectional Manipulator is composed of two bidirectional origami joints with similar tube, thread, knot, and pouch architectures (Figure 9). Figure 9a illustrates the direction of movement for the manipulator. Starting from the intermediate figure where both origami joints are in the unfolded state with $\varphi_{1}$ and $\varphi_{2}$ close to $180^{\circ }$, the manipulator can reversibly fold either to the left or right, as shown in the left and right figures.

Figure 9b displays the trajectory of the motion capture marker on the corner of the output plate of origami 2. The two folded states and the unfolded state are marked. The limiting folding angle of origami 1 is greater (less extreme) than that of origami 2 in both folding directions. This is due to the discrepancy in their loading: the motion performance of origami 1 is reduced by the additional weight of origami 2, whereas origami 2 does not carry a load. Both joints have a smaller range of motion (larger value of φ in the maximally folded state) when integrated into the manipulator than in isolation, but the manipulator is still able to fold into itself almost completely in both directions.

In principle, by coordinating the timing of the pouches’ inflation and deflation, the manipulator could achieve a more consistent and predictable motion during the unfolding process. However, in this experiment, on each side of the origami joints, both pouch actuators are connected to the same solenoid using a three-way connector (Figure S1). Thus, the pouch pressure is identical between them, but due to their different loading and physical position, they do not always inflate equally. This creates an interesting hysteretic effect in the trajectories shown in Figure 9b. When unfolding from either of the two fully folded states, both joints fold evenly, and the manipulator extends along a smooth arc. However, when folding from the unfolded state, origami 1 completes its fold earlier than origami 2, producing a more complex path. This behavior is additionally plotted as a function of time in Figure S4.

Overall, the simulation shape differs from the experimental results, but the manipulator’s range of motion remains similar. The reason for the variation is that the origami joints have different maximum possible folding angles due to factors such as the load they need to carry and their position in space. If the movement of the joints in different stages is crucial for a specific application, it is possible to utilize additional control valves. By employing separate control valves for each pouch, it becomes feasible to independently control the inflation and deflation stages of the pouches, allowing for more precise and customized manipulator movements.

### 4.2. Self-Lock Manipulator

The Self-Lock Manipulator shown in Figure 10 is constructed identically to the Bidirectional Manipulator but constructed from the original Self-Lock Joint, rather than the bidirectional joint with threads and tubes. As a result, it can fold in only one direction.

The trajectory of the motion capture marker on the corner of the output plate of origami 2 in the Self-Lock Manipulator is shown in Figure 10d. The trajectories exhibit hysteresis similar to that of the Bidirectional Manipulator. The trajectory during the first half of unfolding is somewhat inconsistent due to the position of the pouch and the space restrictions for inflation. Afterward, when the pouches are no longer confined, their inflation and deflation synchronize in the middle of the unfolding motion, and the motion becomes more consistent. This synchronization allows for a smooth and controlled unfolding of the manipulator until it reaches the unfolded state. Origami 2 unfolds completely, with $\varphi_{2}=180^{\circ }$, whereas origami 1 unfolds only to an angle of $\varphi_{1}=157.8^{\circ }$ due to the weight of origami 2. These trajectories are shown as a function of time in Figure S5.

By combining a reduced central angle in the Self-Lock Manipulator and considering the extensibility of the organza fabric, it is possible to create a strong and stable structure. The smaller central angle helps overcome the extensibility of the fabric, ensuring that the manipulator maintains its desired configuration even when subjected to heavy loads or external forces. This feature is particularly advantageous in applications where stability and resistance to configuration changes are crucial, allowing the manipulator to perform reliably and effectively under challenging conditions.

## 5. Limitations and Future Work

In future work, a study is required to investigate the joint’s moments using various fabrication methods in order to optimize its performance. Additionally, different origami models can be employed to achieve diverse motions for various applications. Thus, the development of a program that can take user-defined positions for robot exploration and suggest suitable origami models capable of achieving the desired motion is essential. Furthermore, a control scheme needs to be established for the joint to manipulate its configuration based on the desired end-effector position of the manipulator, enabling it to perform specific tasks effectively.

## 6. Conclusions

Flat-foldable origami structures offer rapid and cost-effective fabrication benefits. As a result, replacing traditional robotic parts with these structures could bring about similar advantages in robotics. The previously proposed theoretical Self-Lock Origami, which exhibits partial flat-foldability due to its rigid plates, provides a significantly faster rotational motion compared with a simple fold (traditional revolute joint) with the same actuator. In this study, both bidirectional and one-directional origami joints are fabricated based on the theoretical Self-Lock Origami via 3D printing with an integrated fabric layer. We successfully resolved the semi flat-foldability issue and doubled the range of motion in the bidirectional origami joint. Furthermore, the joints exhibit the capability to handle heavy loads. We compared the performance of origami joints with different plate thicknesses, wedge lengths, spacing between the plates, fabric types, and central angles. We observe that an increase in plate thickness leads to a decrease in the rotational range. On the other hand, increasing the wedge length enhances the soft space between the plates, resulting in an improved range of motion. Increasing the spacing between the plates has a similar effect. However, an excessively large soft area between the plates weakens the structure, leading to a significant reduction in the range of motion. Among the tested fabrics for the soft joint between the origami plates, organza demonstrates promising results due to its flexibility and durability. Also, our results indicate that higher fabric stiffness reduces the range of motion. We developed bidirectional and one-directional manipulators as a proof of concept. Overall, this research presents a promising future for robotics with 3D-printed origami joints that offer both flat-foldability and exceptional kinematic performance.

## Data Availability

The data presented in this study are available from the corresponding author upon request.

## Supplementary Materials

The following supporting information can be downloaded at https://www.mdpi.com/article/10.3390/mi14081649/s1, Section S1: Description of control electronics; Figure S1: Diagram of control electronics; Figure S2: Forces study of the Self-Lock Origami; Figure S3: Temporal perspective on Figure 8; Figure S4: Temporal perspective on Figure 9; Figure S5: Temporal perspective on Figure 10; Table S1: Logic table for control electronics.
