# Supplementary material for: Three-Dimensionally Printed Self-Lock Origami: Design, Fabrication, and Simulation to Improve Performance of Rotational Joint
Source: Micromachines (Basel). 2023 Aug 21;14(8):1649. doi: 10.3390/mi14081649 (PMC10456827; doi:10.3390/mi14081649)
Supplement: Supplementary file 1 [file micromachines-14-01649-s001.zip › Supplementary Materials.pdf]

Supplementary Materials: Three-Dimensionally Printed Self-Lock Origami: Design, Fabrication, and Simulation to Improve Performance of Rotational Joint

Samira Zare [1,*], Alex Spaeth [1], Sandya Suresh [2] and Mircea Teodorescu [1,*]

Section S1 Control Electronics

A bang-bang control system has been developed to activate the Self-Lock origami joint (Figure S1A) using pressure sensors (system safety) for feedback. The blue lines represent the flow of air in the system (connecting pump output to the pouches), while the red lines transmit digital signals. Each origami joint requires two pouch motors for downward and upward movements. As shown in Figure S1A, four 3-way mini Solenoid with air gas valves (Sol 1- Sol 4) are used to activate the pouches (Figure S1B(a,c)). Sol 1 controls the miniature 5V miniature pump/vacuum device (B078H8V563) for inflating any of the pouches, while Sol 4 manages the deflation of the pouches. To prevent any potential damage to the system, a valve check and valve control (regulator) are installed between the pump and Sol 1. This setup ensures proper functioning and protection of the system. Sol 2 and Sol 3 are responsible for selecting which pouch to inflate and deflate, respectively.

Two versions of modular PCB boards with or without a pressure sensor circuit (Figure S1B(b)) have been developed. Figure S1B(c) illustrates the installation of a Nano Arduino microcontroller, TIP120 transistor, 1KΩ resistor, and 1N4004 diode on the PCB board to enable the control of a solenoid. In the system, four of these PCB boards, each with its respective installation, are utilized along with a shared microcontroller to control the four solenoids. We measured the time required to fully inflate the pouch motor and utilized this information to change and estimate the status of the pouches. The pressure sensors (Adafruit MPRLS Ported Pressure Sensor) can be utilized to verify the status of the pouches (Figure S1(d)) before inflating or deflating them, thereby preventing any potential damage to the pouches. Additionally, a protective deflation procedure is implemented before activating any of the pouches based on user inputs to avoid issues. If the system attempts to inflate a fully inflated pouch, the pressure sensor detects it, triggering the protective deflation procedure to be executed first.

Initially, the time 't' required for the pump to fully inflate the pouch is measured (Figure S1B(a)). Then, based on the digital I/O inputs (ON/OFF switches) from the user and microcontroller programming, the values of digital pins D1-D4 change to HIGH/LOW (on/off). The corresponding status of each pouch based on the I/O switch values can be found in Figure S1. Switch 1 input activates pouch 1. When switch 1 is turned on and switch 2 is off, pouch 1 inflates while pouch 2 deflates. The digital pins D1-D4 are set to the 'on' state. The 'off' values of pins D1 and D4 indicate that the airflow is outside of the system and would not affect the status of the pouches. After time 't', D1 and D4 are switched to the 'off' state to maintain pouch 1 in an inflated state and pouch 2 in a deflated state, respectively. Switch 2, on the other hand, corresponds to pouch 2 activation. When switch 1 is off and switch 2 is on, D1 and D4 are turned on for time 't', while D2 and D3 are turned off to inflate pouch 2 and deflate pouch 1. To continuously switch between inflating and deflating pouch 1 and pouch 2, both switch 1 and switch 2 should be turned on. Therefore, the microcontroller outputs 'on' for D1 and D4 digital pins. The values of D2 and D3 are the same and should be alternated (on/off) every 't' time. The last case occurs when both switch 1 and switch 2 are off. In this case, D1 is off, D4 is on, D3 is on for time 't', and then is off, causing both pouches to deflate. The value of D4 can be changed at the time 't' from on to off to stop the deflation. The D2 signal is irrelevant in this case because D1 is off and the pump air is outside of the system.

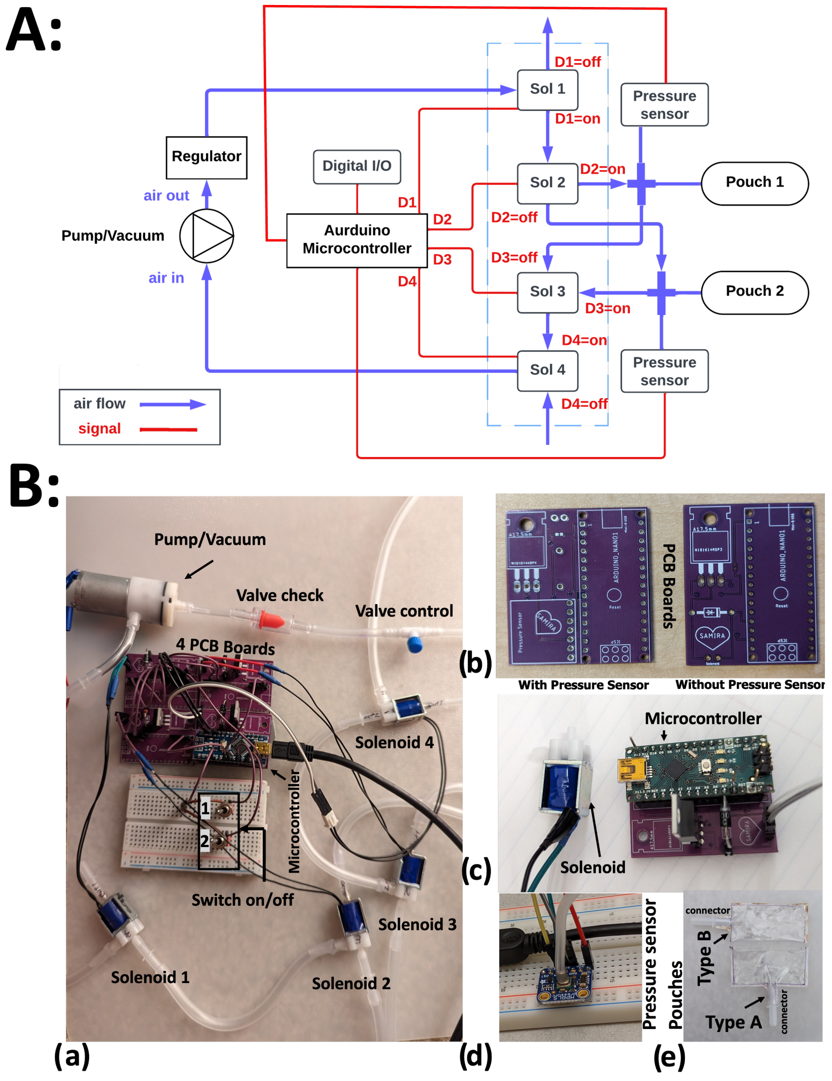

Figure S1. A: The control diagram of the system. The origami joint consists of two pouches, namely pouch 1 and pouch 2, which need to be activated. To achieve this, four solenoids (Sol1 - Sol4) along with a microcontroller are employed. A regulator is utilized to control the air flow, while a digital I/O interface receives inputs from the user to activate the pouches. The Sol 1 is responsible for controlling the pump, while Sol 4 manages the vacuum. The blue arrow indicates the direction of air flow, while the red line represents the digital signal. When the Sol 1 and Sol 4 signals are off (LOW), it signifies that the air flow is outside the system, and vice versa. Similarly, Sol 2 and Sol 3 are utilized to control the inflation and deflation of the pouches, respectively. For instance, when Sol 1 receives a signal of D1 = on and Sol 2 receives a signal of D2 = on, it results in the inflation of pouch 1. Pressure sensors are integrated into the system to verify the status of the pouches and protect the system. **B:** (**a**) The devices utilized in the activation system include a pump/vacuum for pouch inflation and deflation, valve check and valve control for system protection, Switch 1 and Switch 2 as digital I/O interfaces to receive user inputs, four solenoids, their respective PCB boards, and a microcontroller for controlling pouch activation. (**b**) Two types of PCB boards have been designed to control the solenoids: one with pressure sensor circuits and one without. (**c**) The necessary devices, including a microcontroller, transistor, diode, and resistor, are installed on the PCB board to control a solenoid. (**d**) A pressure sensor is employed to verify the status of the pouches. (**e**) Type A pouches feature connectors at the bottom, while Type B pouches have connectors at the side. The development of Type B pouches aims to prevent interference between the joint's connector and tube with the rotational motion of other origami components.

Table S1. Pouch motors' status based on digital I/O inputs.

Digital I/O		Pouch status	
**switch 1**	**switch 2**	**pouch 1**	**pouch 2**
on	off	inflated	deflated
off	on	deflated	inflated
on	on	inflated/deflated	deflated/inflated
off	off	deflated	deflated

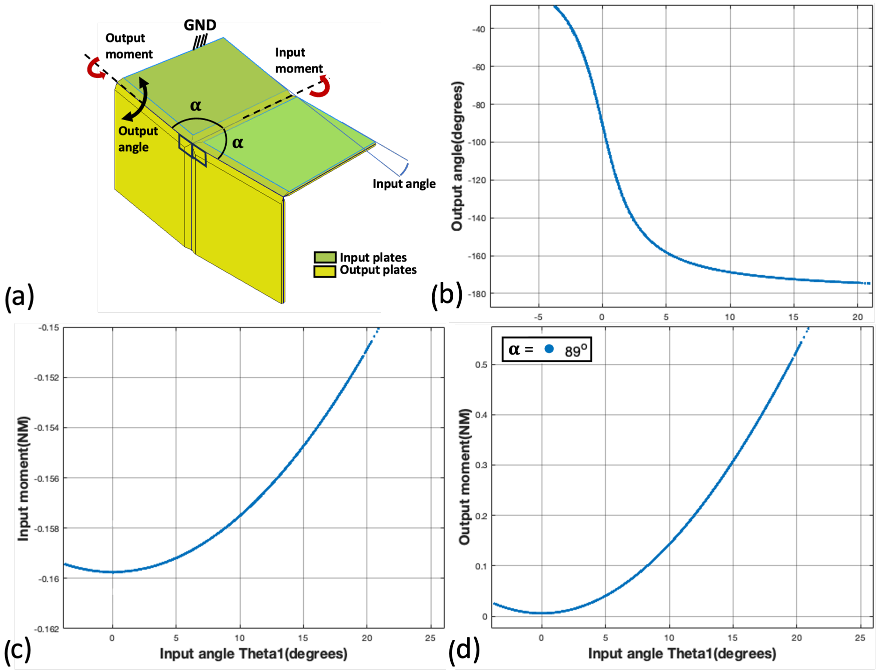

Figure S2. The angles and forces of the Self-lock origami with one-directional movement using the hybrid model: The origami's output angle measurements are used from the experiment, while the equations provided in [1] are utilized to derive the corresponding angles and forces. - a) The origami's structural features include a central angle of 89° and associated parameters. Actuators are positioned on the input plates, with one plate being grounded. The input moment refers to the force exerted on the origami by the actuator attached to the ungrounded input plate. On the other hand, the output moment arises from the force generated by the output plate connected to the grounded plate. A consistent actuator setup is employed for activating the origami in all the experiments. b) Input angle: the supplementary angle between the input plates. The input angle's range is limited due to the actuator's and origami's geometry (one-directional movement). The output plate: the negative angle between the grounded and the output plates. c and d) the origami forces' plots. Despite the inflation of the pouch motor, due to the one-directional configuration of the origami, the left side of Theta1 = 0 degree has smaller forces.

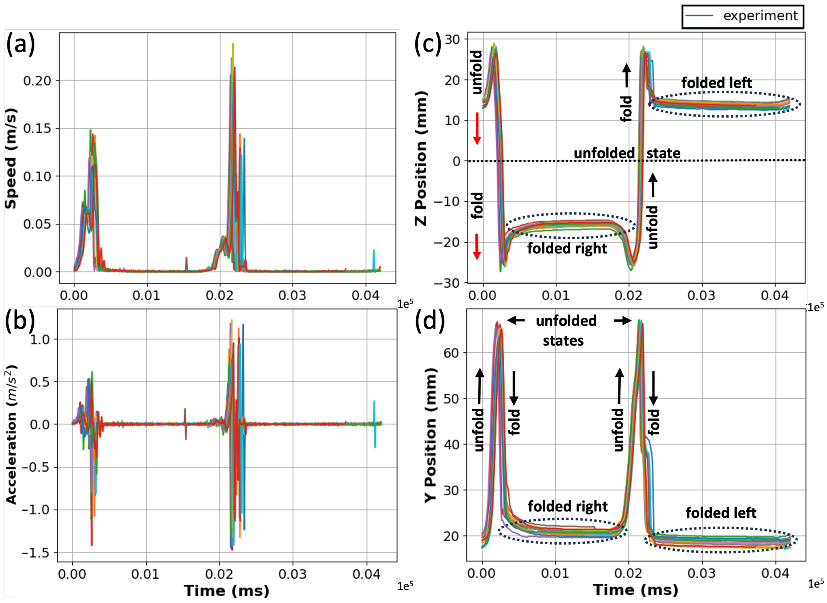

Figure S3. A closer look into the origami marker's a) Speed plot based on time. b) Acceleration plot based on time. c) and d) Y and Z positions based on time. The folded and unfolded states, along with the directions of movement, are demonstrated in parts c and d. In both the folded and unfolded states, the system maintains the pouches inflated for approximately 2e3 milliseconds.

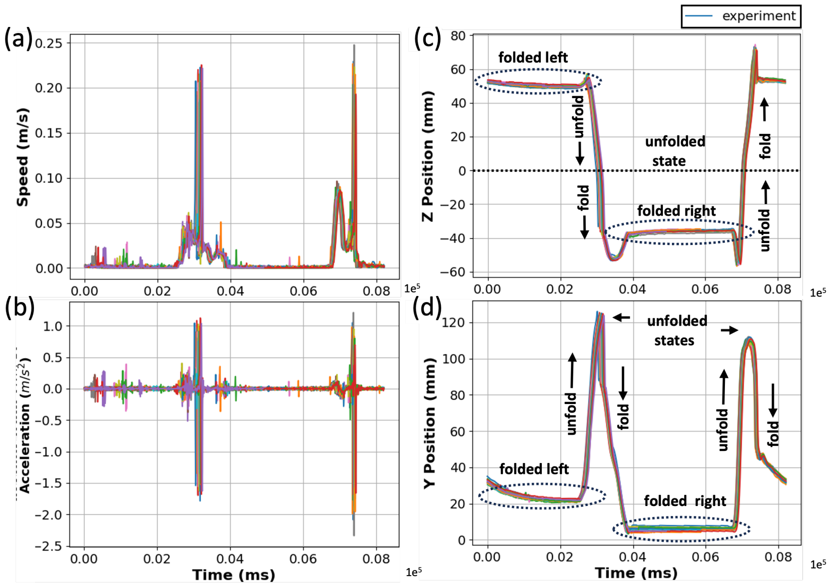

Figure S4. For bi-directional Manipulator's end-effector: The origami marker's a) Speed plot based on time. b) Acceleration plot based on time. c) and d) Y and Z positions based on time. The folded and unfolded states, along with the directions of movement, are demonstrated in parts c and d. In both the folded and unfolded states, the system maintains the pouches inflated for approximately 4e3 milliseconds.

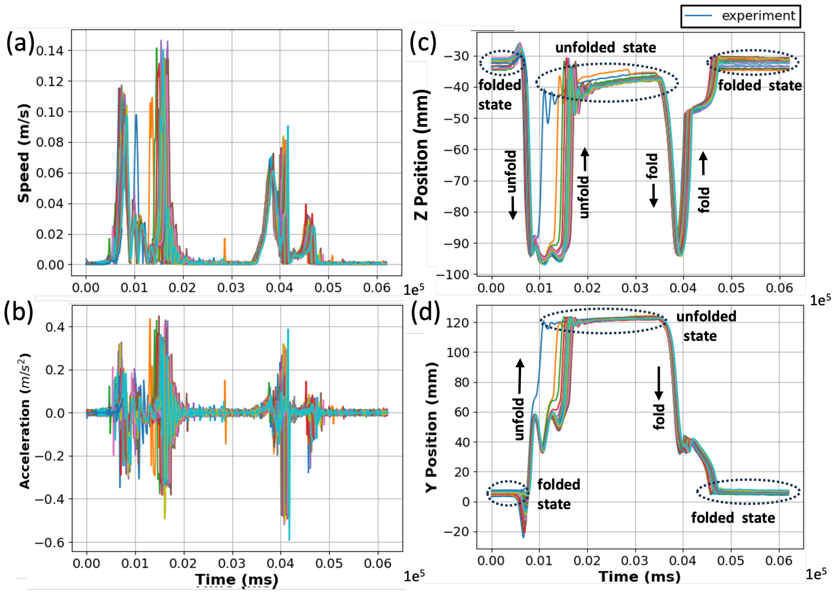

Figure S5. For one-directional Manipulator's end-effector: The origami marker's a) Speed plot based on time. b) Acceleration plot based on time. c) and d) Y and Z positions based on time. The folded and unfolded states, along with the directions of movement, are demonstrated in parts c and d. In both the folded and unfolded states, the system maintains the pouches inflated for approximately 3e3 milliseconds.

References

1. Zare, S.; Spaeth, A.; Suresh, S.; Teodorescu, M. Modular Self-Lock Origami: Design, modeling, and simulation to improve the performance of a rotational joint. *arXiv* **2023**, arXiv:2307.16393.
