# Supplementary figures and images for: Three-Dimensionally Printed Self-Lock Origami: Design, Fabrication, and Simulation to Improve Performance of Rotational Joint
Source: Micromachines (Basel). 2023 Aug 21;14(8):1649. doi: 10.3390/mi14081649 (PMC10456827; doi:10.3390/mi14081649)

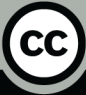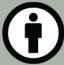

BY

Supplement: Supplementary file 1 [file micromachines-14-01649-s001.zip › Definitions/logo-ccby-eps-converted-to.pdf]

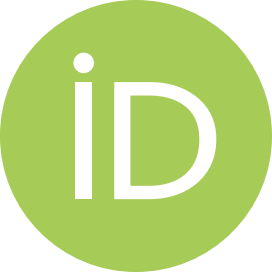

Supplement: Supplementary file 1 [file micromachines-14-01649-s001.zip › Definitions/logo-orcid.pdf]

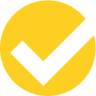

check for  
updates

Supplement: Supplementary file 1 [file micromachines-14-01649-s001.zip › Definitions/logo-updates-eps-converted-to.pdf]

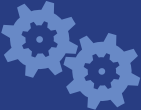

*micromachines*

Supplement: Supplementary file 1 [file micromachines-14-01649-s001.zip › Definitions/micromachines-logo-eps-converted-to.pdf]

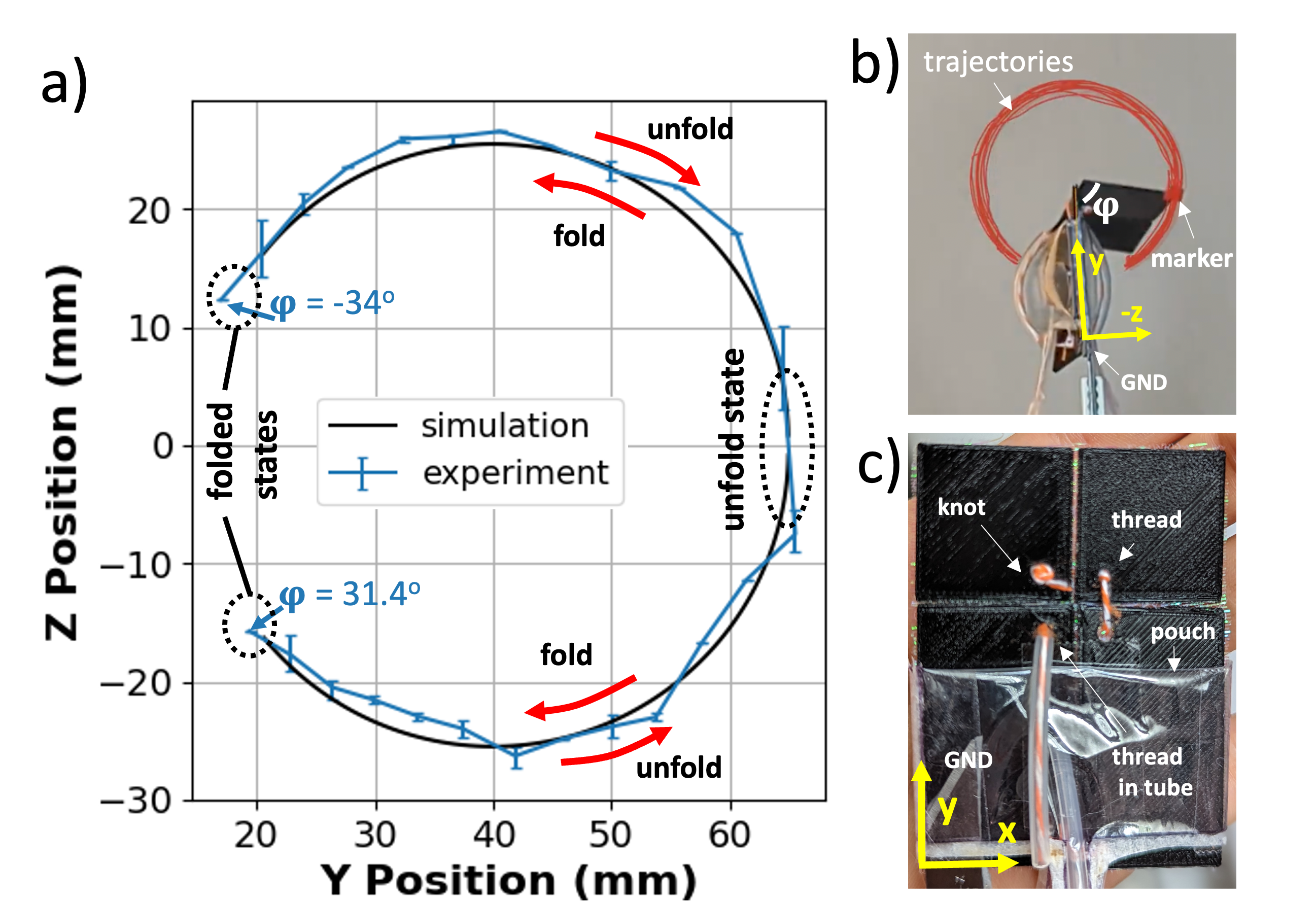

Supplement: Supplementary file 1 [file micromachines-14-01649-s001.zip › images/bi-directional.png]

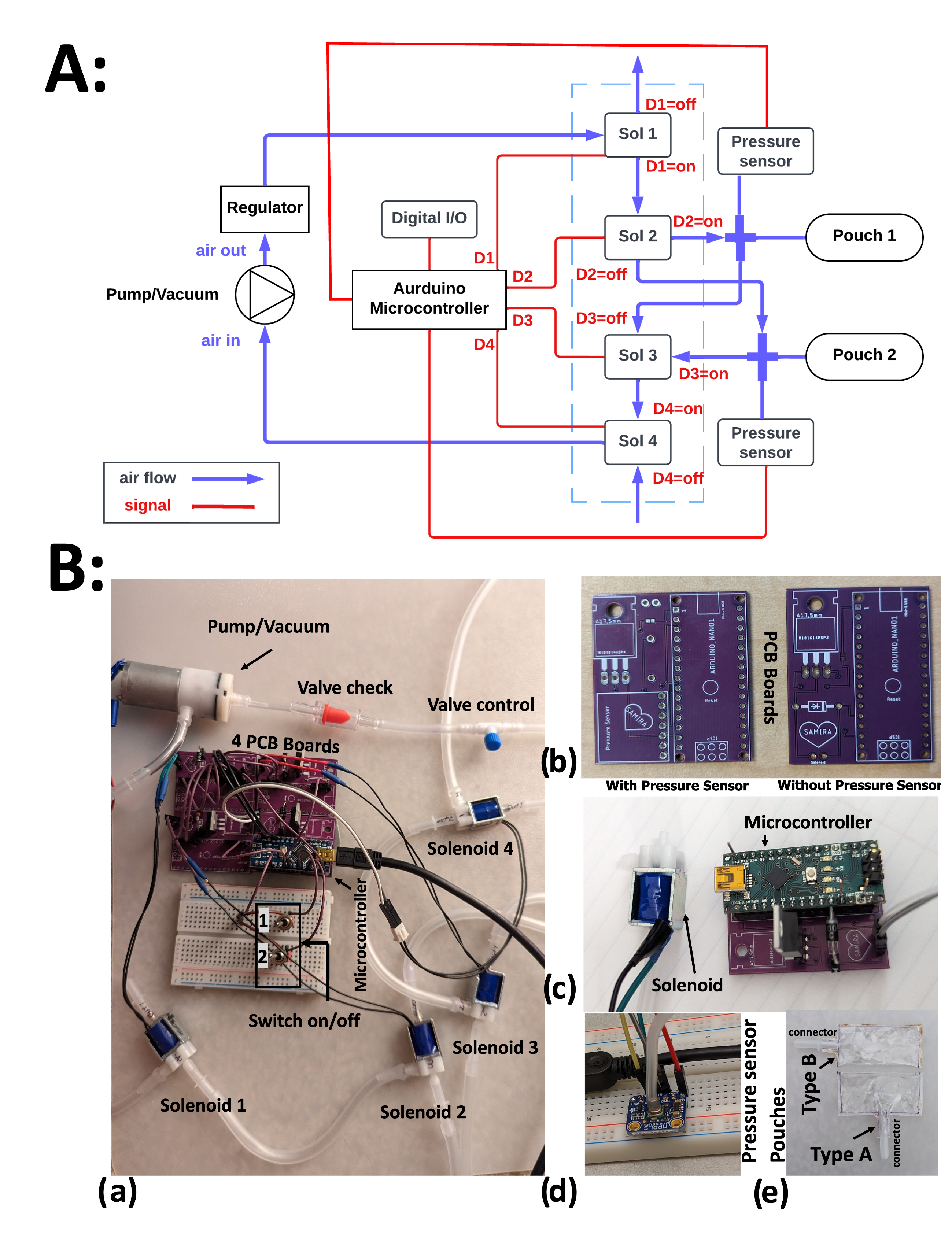

Supplement: Supplementary file 1 [file micromachines-14-01649-s001.zip › images/controll.png]

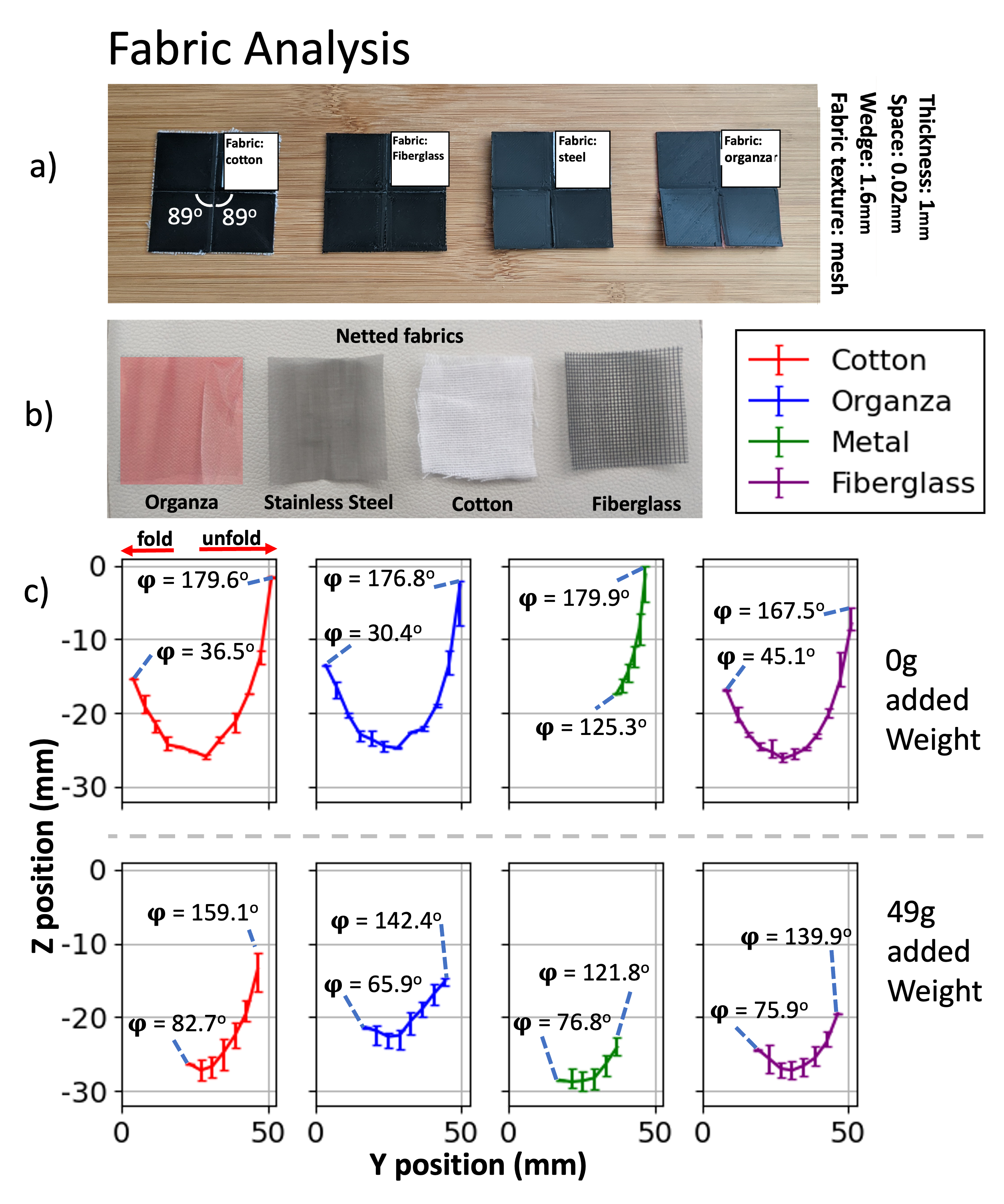

Supplement: Supplementary file 1 [file micromachines-14-01649-s001.zip › images/diffFabric.png]

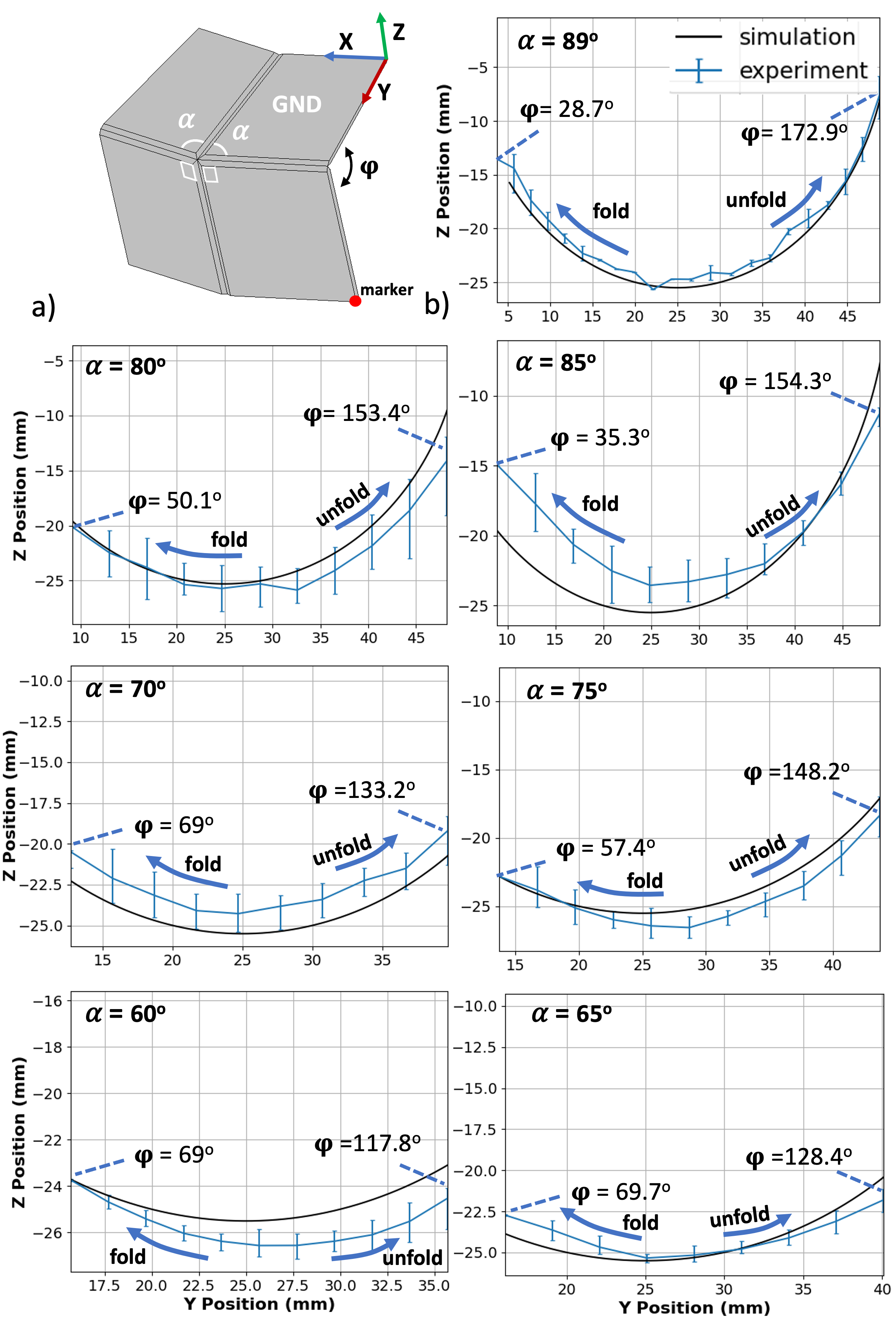

Supplement: Supplementary file 1 [file micromachines-14-01649-s001.zip › images/diffModels.png]

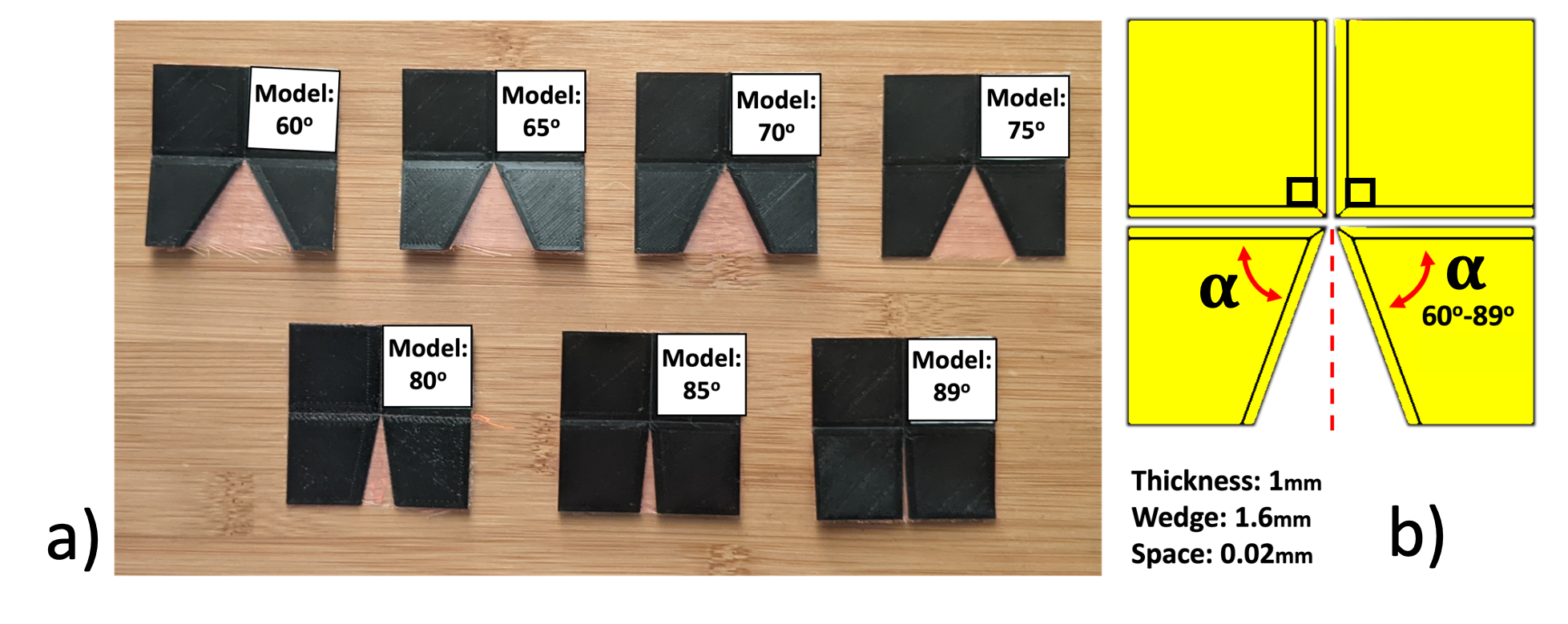

Supplement: Supplementary file 1 [file micromachines-14-01649-s001.zip › images/diffModels1.png]

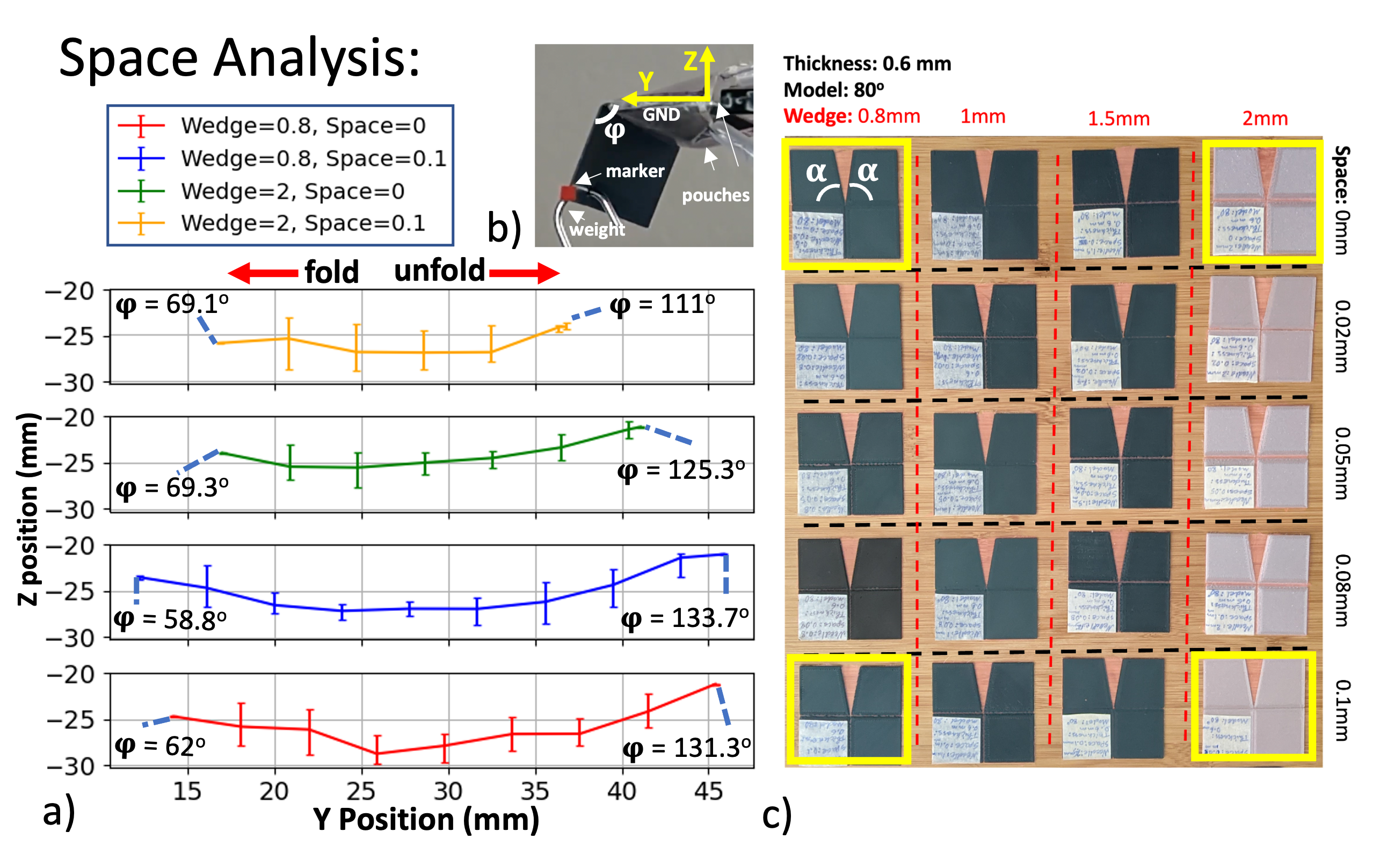

Supplement: Supplementary file 1 [file micromachines-14-01649-s001.zip › images/diffSpace.png]

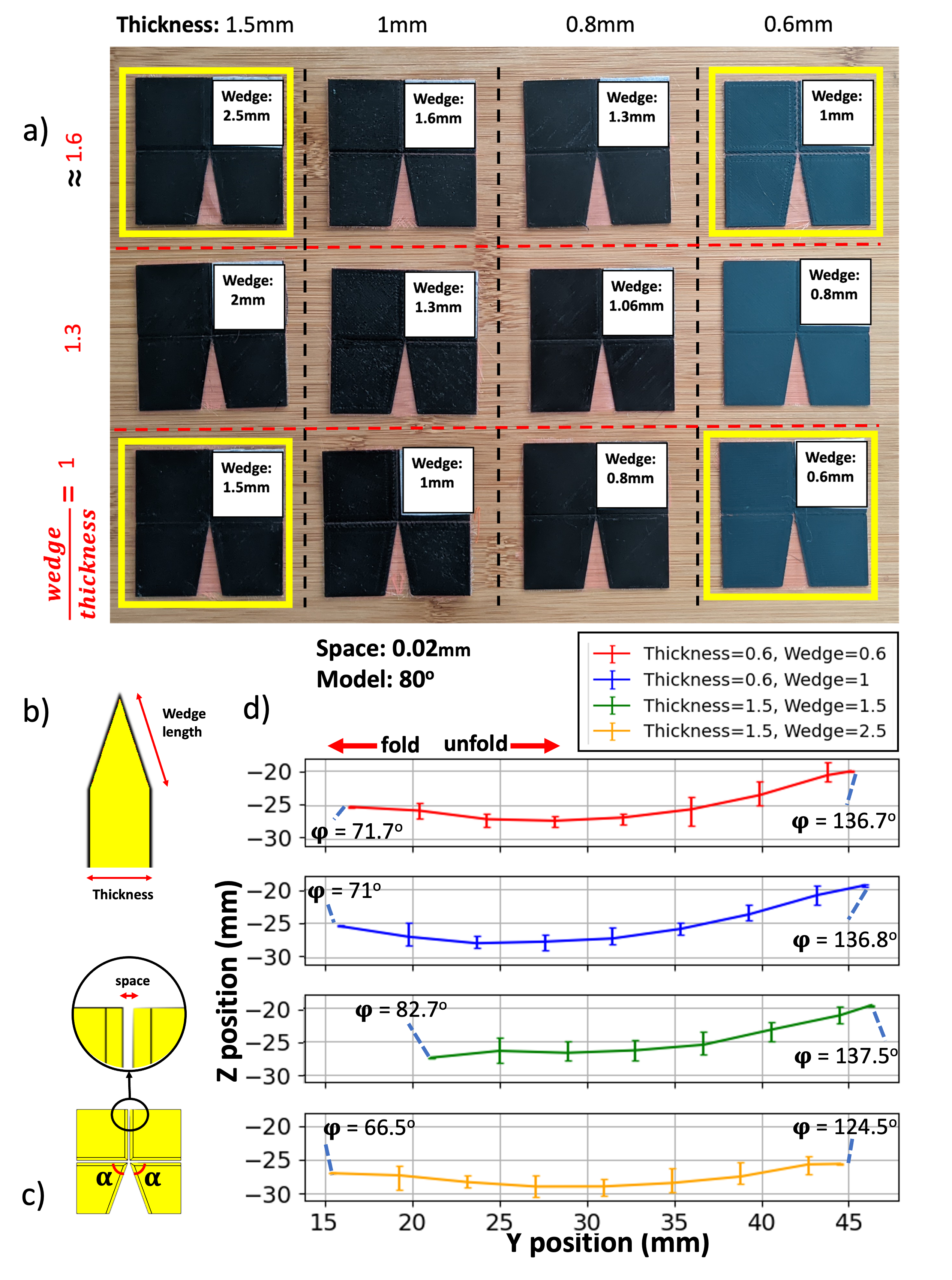

Supplement: Supplementary file 1 [file micromachines-14-01649-s001.zip › images/diffThickness.png]

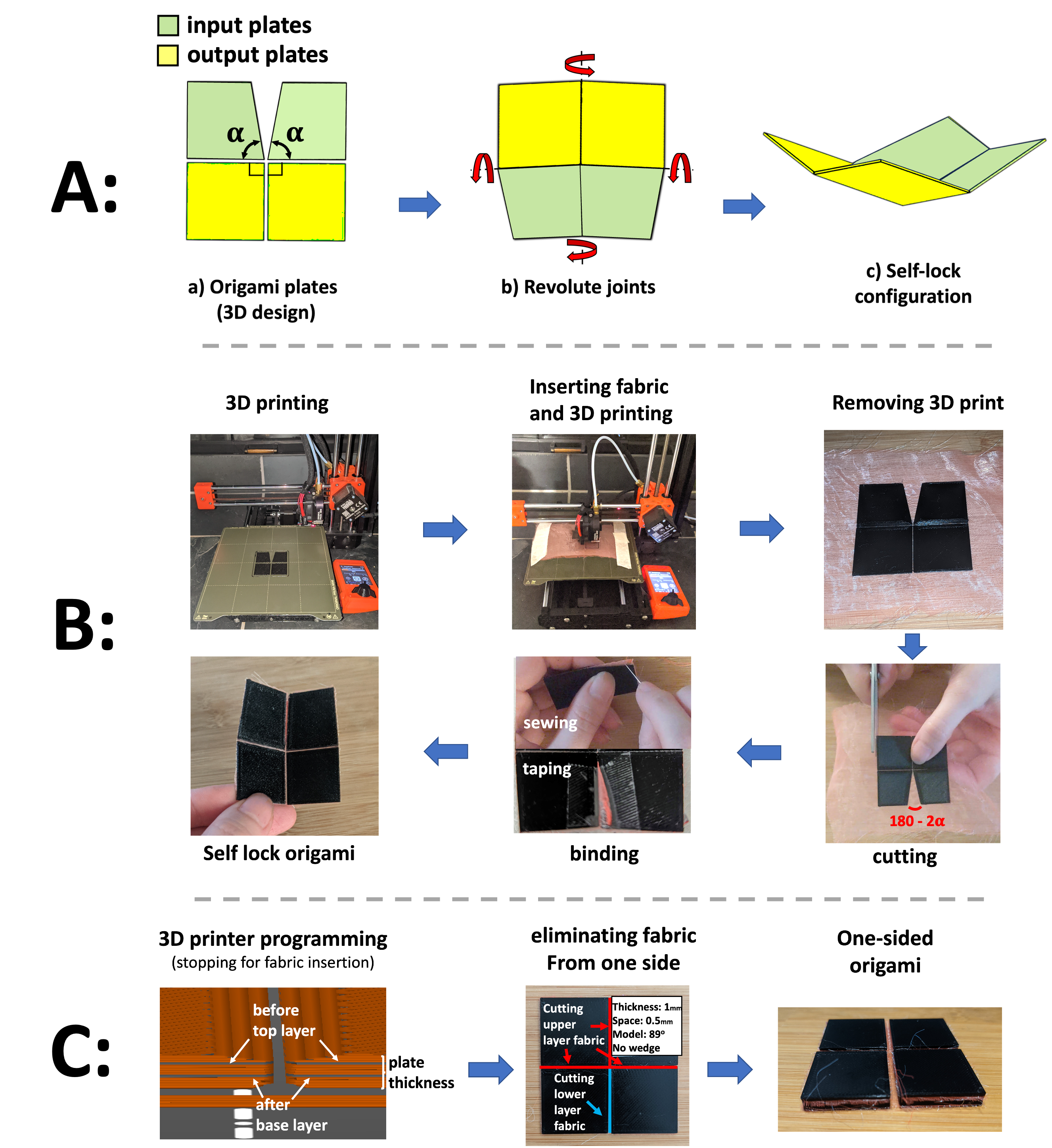

Supplement: Supplementary file 1 [file micromachines-14-01649-s001.zip › images/fabrication.png]

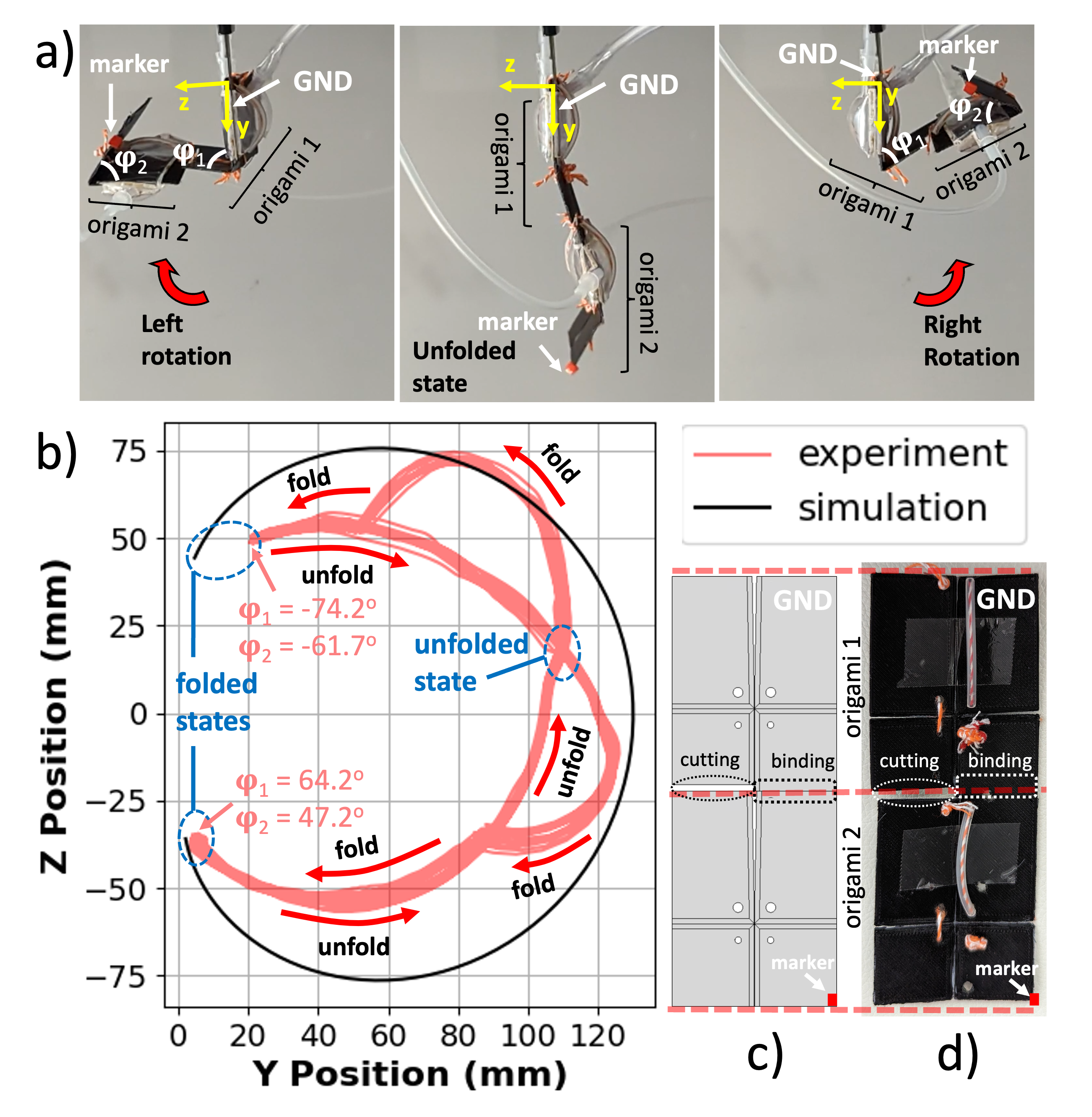

Supplement: Supplementary file 1 [file micromachines-14-01649-s001.zip › images/maniBi-directional.png]

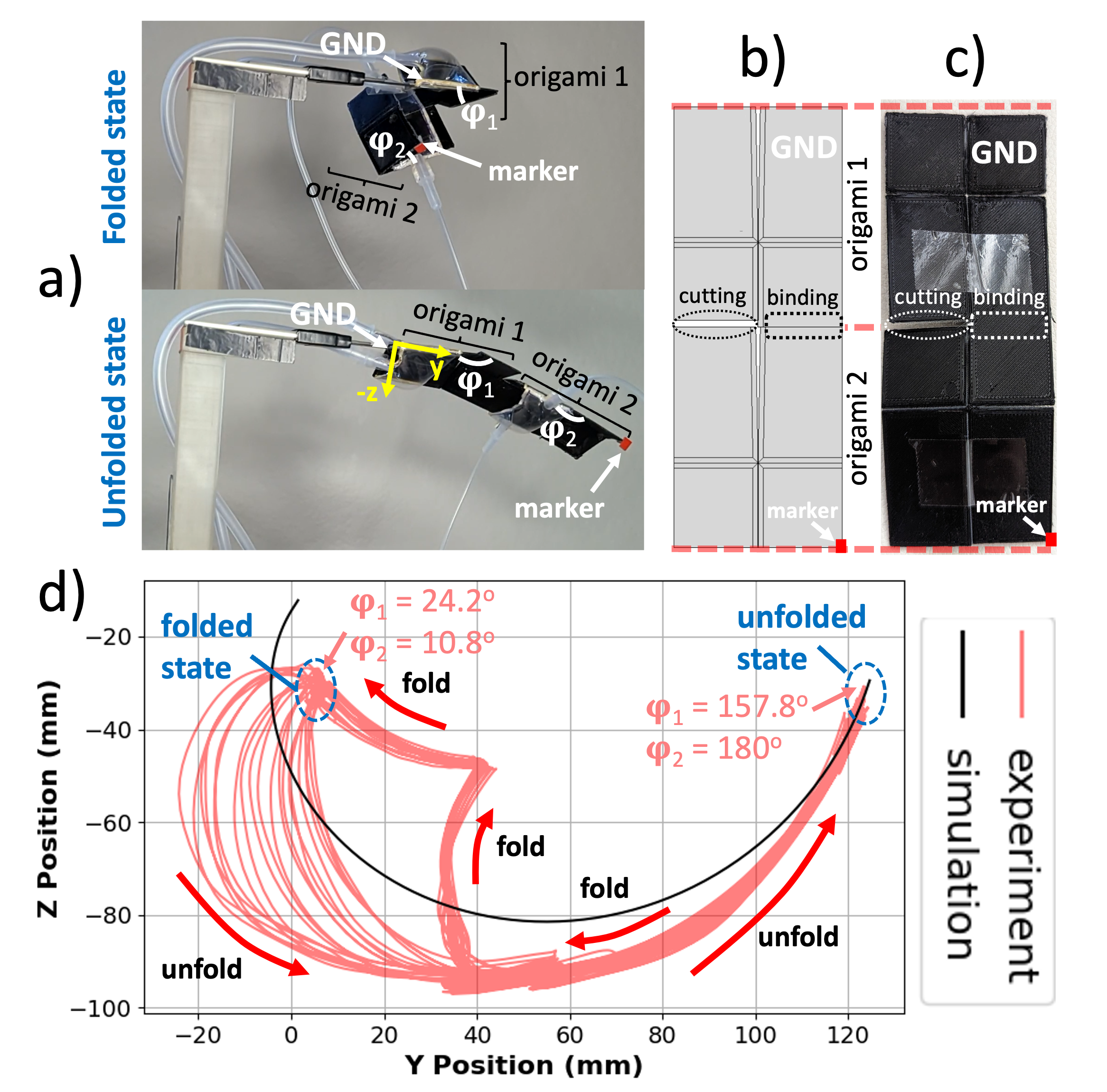

Supplement: Supplementary file 1 [file micromachines-14-01649-s001.zip › images/ManiDirectional.png]

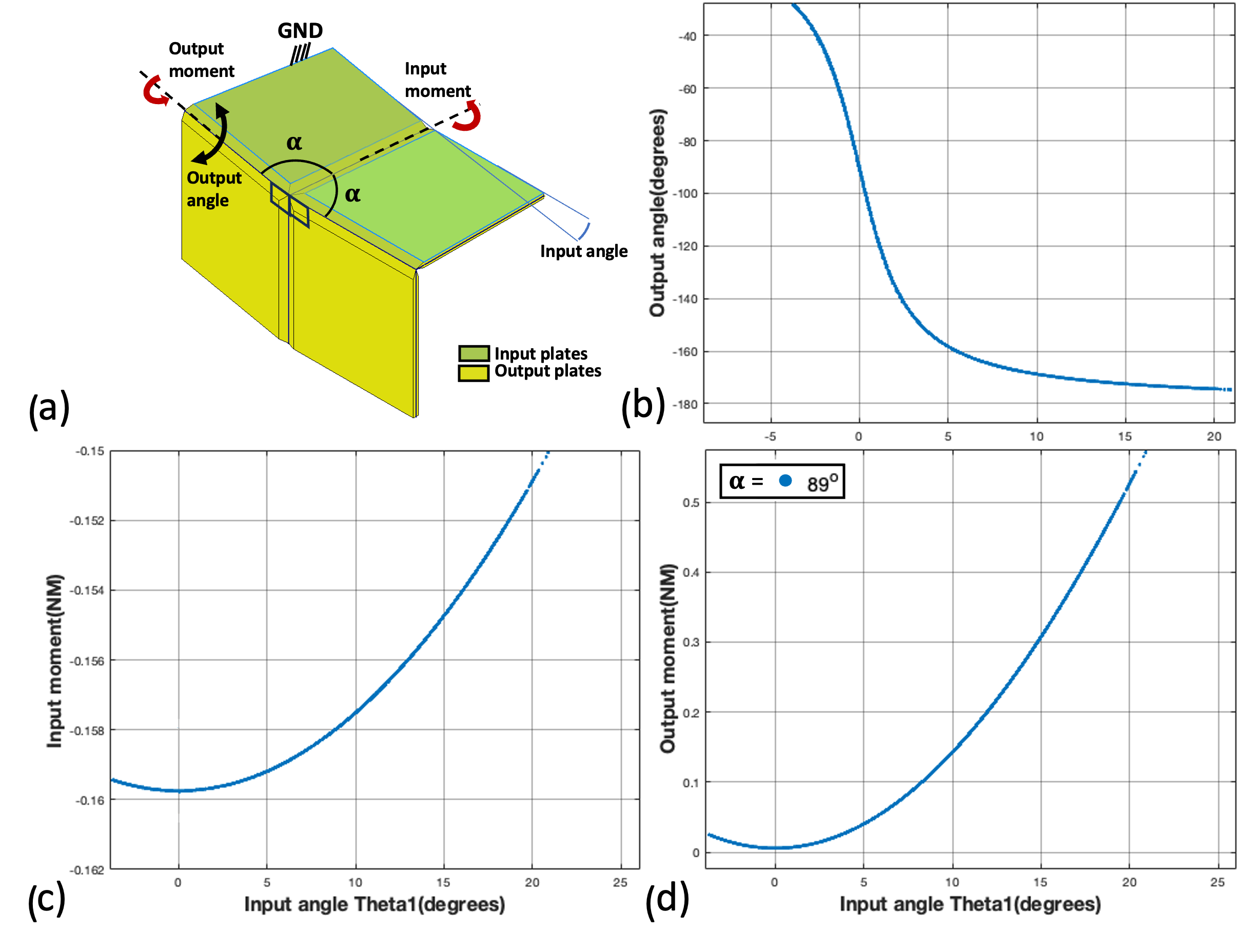

Supplement: Supplementary file 1 [file micromachines-14-01649-s001.zip › images/Moments.png]

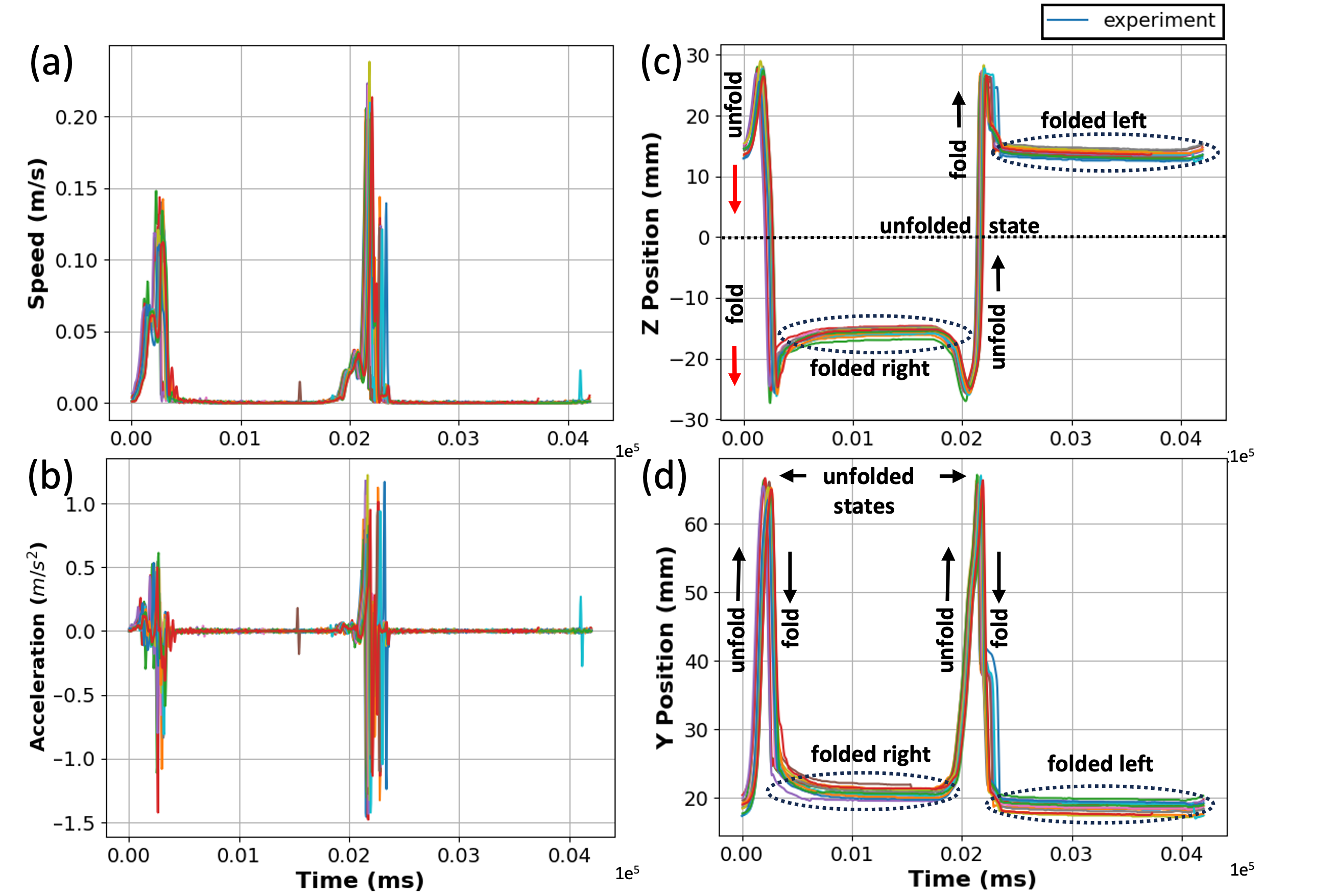

Supplement: Supplementary file 1 [file micromachines-14-01649-s001.zip › images/TimeBiDirectional.png]

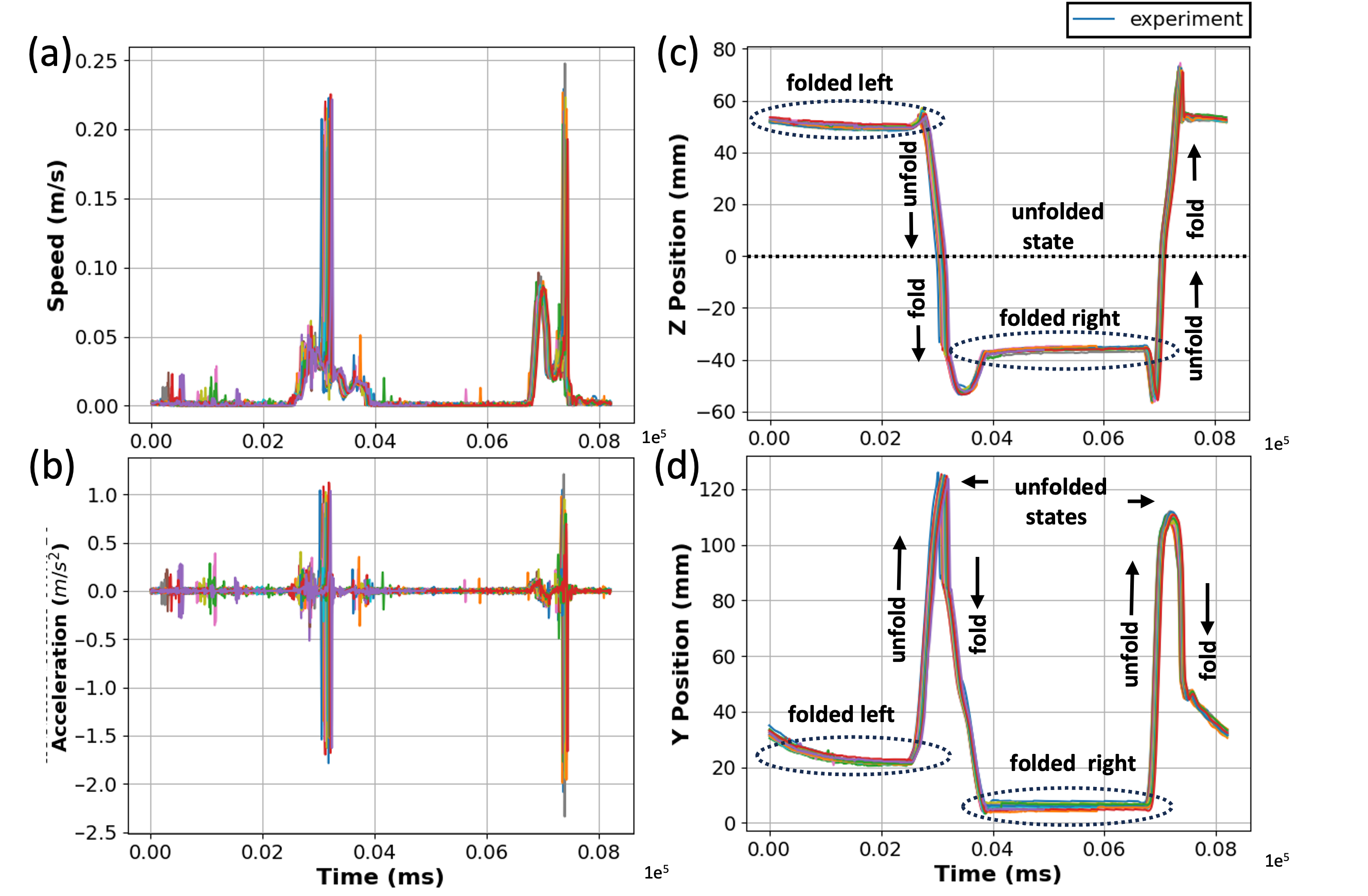

Supplement: Supplementary file 1 [file micromachines-14-01649-s001.zip › images/TimeBiDirectionalMani.png]

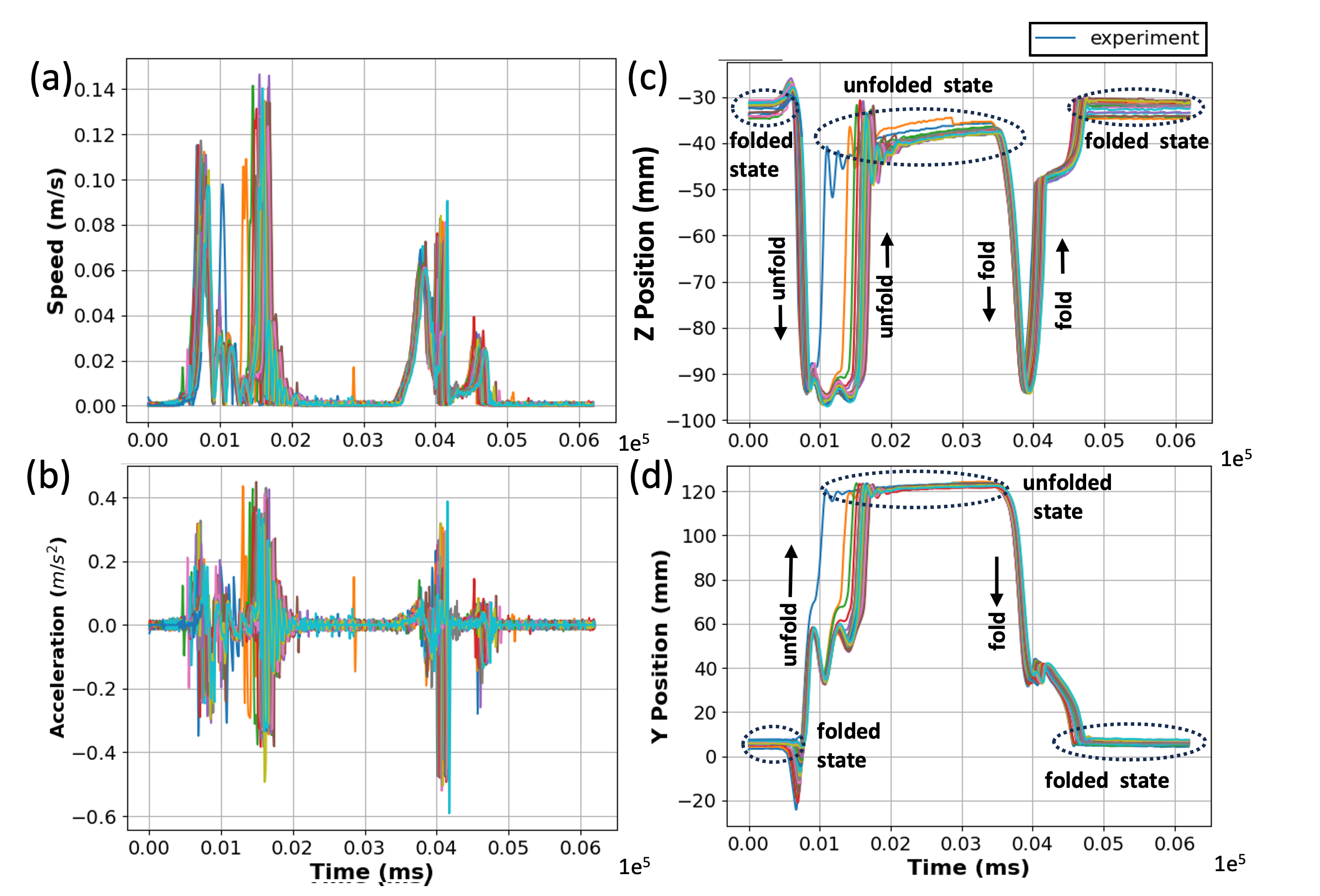

Supplement: Supplementary file 1 [file micromachines-14-01649-s001.zip › images/TimeDirectedMani.png]

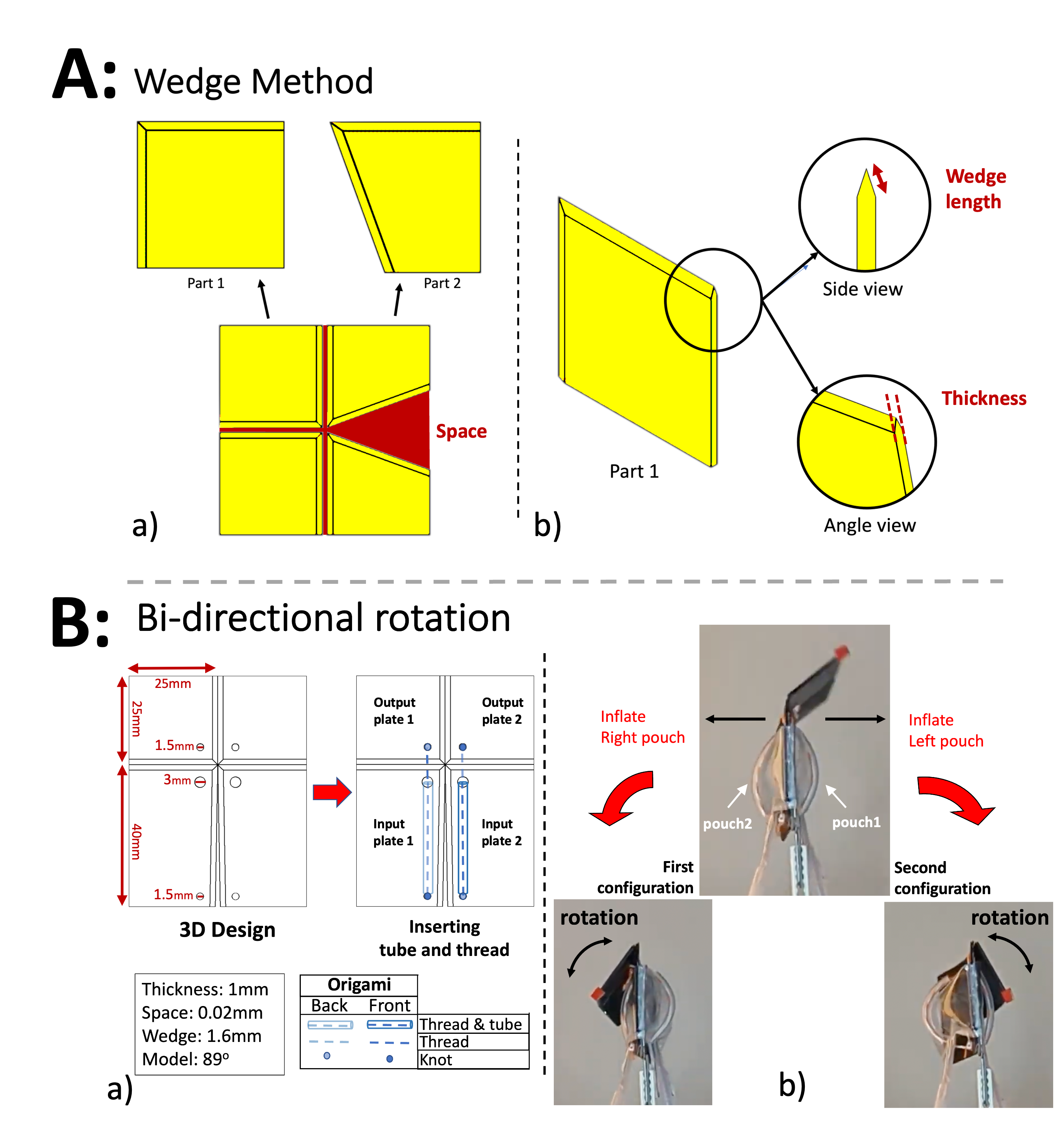

Supplement: Supplementary file 1 [file micromachines-14-01649-s001.zip › images/Wedge.png]
